# Alternative lengthening of telomeres (ALT) cells viability is dependent on C-rich telomeric RNAs

**DOI:** 10.1038/s41467-023-42831-0

**Published:** 2023-11-04

**Authors:** Ilaria Rosso, Corey Jones-Weinert, Francesca Rossiello, Matteo Cabrini, Silvia Brambillasca, Leonel Munoz-Sagredo, Zeno Lavagnino, Emanuele Martini, Enzo Tedone, Massimiliano Garre’, Julio Aguado, Dario Parazzoli, Marina Mione, Jerry W. Shay, Ciro Mercurio, Fabrizio d’Adda di Fagagna

**Affiliations:** 1 IFOM ETS - The AIRC Institute of Molecular Oncology, Milan, Italy; 2 IFOM ETS - The AIRC Institute of Molecular Oncology (Experimental Therapeutics Program), Milan, Italy; 3 Institute of Biological and Chemical Systems – Functional Molecular Systems (IBCS-FMS), Karlsruhe Institute of Technology (KIT), Karlsruhe, Germany; 4 School of Medicine, Universidad de Valparaiso, Valparaiso, Chile; 5 Dipartimento di Oncologia ed Emato-Oncologia, Università degli Studi di Milano, Milan, Italy; 6 Department of Cell Biology, University of Texas Southwestern Medical Center, Dallas, TX USA; 7 Department of Cellular, Computational and Integrative Biology – CIBIO, University of Trento, Trento, Italy; 8 Institute of Molecular Genetics IGM-CNR “Luigi Luca Cavalli-Sforza”, Pavia, Italy; 9 Present Address: The Salk Institute for Biological Studies, La Jolla, CA USA; 10 Present Address: RCSI, Royal College of Surgeons in Ireland, Department of Chemistry, Dublin, Ireland; 11 Present Address: Australian Institute for Bioengineering and Nanotechnology, The University of Queensland, St Lucia, QLD Australia

**Keywords:** Long non-coding RNAs, Bone cancer, DNA damage response, Homologous recombination, Stalled forks

## Abstract

Alternative lengthening of telomeres (ALT) is a telomere maintenance mechanism activated in ~10–15% of cancers, characterized by telomeric damage. Telomeric damage-induced long non-coding RNAs (dilncRNAs) are transcribed at dysfunctional telomeres and contribute to telomeric DNA damage response (DDR) activation and repair. Here we observed that telomeric dilncRNAs are preferentially elevated in ALT cells. Inhibition of C-rich (teloC) dilncRNAs with antisense oligonucleotides leads to DNA replication stress responses, increased genomic instability, and apoptosis induction selectively in ALT cells. Cell death is dependent on DNA replication and is increased by DNA replication stress. Mechanistically, teloC dilncRNA inhibition reduces RAD51 and 53BP1 recruitment to telomeres, boosts the engagement of BIR machinery, and increases C-circles and telomeric sister chromatid exchanges, without increasing telomeric non-S phase synthesis. These results indicate that teloC dilncRNA is necessary for a coordinated recruitment of DDR factors to ALT telomeres and it is essential for ALT cancer cells survival.

## Abstract (web summary)

ALT cells use an alternative lengthening mechanism of telomeres and bear telomeric DNA damage with increased levels of damage-induced long non-coding RNA. Here the AUs show that antisense oligonucleotides (ASO) targeting such RNAs can induce ALT cancer cells selective cell death.

## Introduction

Telomeres are nucleoprotein structures characterized by repetitive arrays of TTAGGG DNA, located at chromosome termini. They prevent recognition of chromosome ends as damaged DNA, inhibit local DNA damage response (DDR) activation, and protect genome integrity^1^. Telomeric dysfunction, caused by shortening, deprotection or damage, and by DNA replication stress, triggers focal accumulation of activated DDR factors at telomeres, known as telomere dysfunction-induced foci (TIF). Shortening of telomeres with each cell division, caused by the “end replication problem” and nucleolytic activities, eventually results in loss of protection, DDR activation and appearance of TIFs, causing cellular senescence or apoptosis^2–4^. Cancer cells activate telomere maintenance mechanisms to counteract replication-driven telomere shortening and gain replicative immortality. Most often this is achieved through the re-expression of telomerase, an enzyme that catalyzes the addition of telomeric DNA repeats^5^, but 10–15% of cancers use alternative lengthening of telomeres (ALT), a homology-dependent repair (HDR) process that results in telomere lengthening. ALT is common in osteosarcomas^6^ and glioblastomas (GBM)^7^, and no targeted treatment is available yet.

Telomeres of ALT cells display spontaneous TIFs^8^ and commonly colocalize with promyelocytic leukemia (PML) nuclear bodies, forming so-called ALT-associated PML bodies (APBs)^9^. APBs often contain DDR proteins^10^, and are proposed to be the location of telomere elongation in ALT cells^11^. Telomeric damage spurs long-range movement and clustering of ALT telomeres^12^ and can be repaired by the HDR process known as break-induced replication (BIR), which, at ALT telomeres, is initiated by strand invasion promoted by RAD52 and/or RAD51AP1^11,13,14^. This process leads either to telomere lengthening, which is dependent on Polδ accessory subunit POLD3^15^, or to telomeric sister chromatid exchange (T-SCE)^16^, which is non-productive^17^. These aberrant repair processes generate unique telomeric structures including discontinuous DNA and extrachromosomal circles^18,19^, in particular partially single-stranded C-rich telomeric DNA circles called C-circles^20^, whose detection and quantification has become a key determinant for ALT cell identification. Due to this recombinogenic nature of ALT telomeres, they are often long and heterogeneous in length^21^.

Telomeres are fragile chromosomal sites because they are inherently difficult to replicate and are thus prone to DNA replication stress^22^. Telomeric replication stress is elevated in ALT cells^23^ and initiates the DDRs that lead to BIR-mediated lengthening^24–26^, underlining the importance of telomeric replication stress to this pathway. Difficulties in replication of telomeres lie partially in their potential to form secondary structures, including G-quadruplexes (G4s)^27^, and RNA-DNA hybrids formed by transcription of the telomeric RNA TERRA from subtelomeres^28^, both of which are more abundant in ALT cells than in non-ALT cells^29–31^. Another feature of ALT telomeres is the presence of high amounts of telomeric variant repeats, degenerate sequences disrupting the binding of shelterin proteins and recruiting recombinogenic factors^32–34^. Altered telomeric heterochromatin appears to be a component of the ALT pathway, as inactivating mutations in the cooperating histone chaperones ATRX and DAXX are associated with ALT^35,36^ where they contribute to increased sister chromatid cohesion and multiple ALT hallmarks including APBs, C-circles, T-SCE, as well as telomere lengthening^37–39^. ATRX and DAXX deposit histone variant H3.3 at telomeres^40^, and favor macroH2A1.2 deposition during replication stress to promote telomere stability^41^. Similarly, ALT phenotypes are induced upon depletion of the two isoforms of the histone chaperone ASF1, ASF1a, and ASF1b, in cells with long telomeres^42^. It is thought that ASF1 silencing leads to ALT activation through telomeric replication stress induction^42^, suggesting that altered telomeric heterochromatin contributes to replication stress, and highlighting the mechanistic link between DNA replication stress and ALT enforcement.

Replication stress involves stalling or slowing of replication forks, which triggers a response to protect the affected fork in order to allow for repair and replication restart. If not adequately repaired, forks can collapse and can only be rescued by HDR processes such as BIR^43,44^. Tight control of replication stress levels has recently emerged as crucial in the ALT pathway. Both decreasing and increasing telomeric replication stress levels in ALT cells appears to be detrimental, resulting in diminished ALT activity and progressive telomere shortening, or increased ALT activity and hyperrecombination resulting in cell death, respectively^26,29,45^.

Throwing off the balance of replication stress levels is at the core of many strategies proposed to specifically target ALT cells. For example, telomeric replication stress augmentation by the telomere-specific G-quadruplex ligand Tetra-Pt(bpy) induces telomeric DNA damage, exacerbates ALT phenotypes, and causes ALT cells death^46^. Similarly, depletion or inhibition of telomere localization of FANCM, which resolves RNA-DNA hybrids at telomeres, results in toxic levels of RNA-DNA hybrids^45^. Other approaches to specifically target ALT cells have also been reported, including depletion of testis-specific Y-encoded-like protein 5 (TSPYL5) that leads to ALT-specific degradation of POT1^47^, and inhibition of lysine acetyl transferase^48^. It is tempting to consider manipulation of DDR signaling as a strategy to skew the balance of repair towards toxicity. Indeed, inhibition of DDR, through inhibition of ATR, has been proposed to induce ALT-specific cell death^49^, although specificity has been questioned^50–52^.

Although ALT-specificity of global DDR inhibition remains unclear, telomere-specific DDR inhibition could still result in selective targeting of ALT cells. Sequence-specific manipulation of DDR was recently made possible by the discovery of damage-induced RNAs, and their role in the DDR. We have recently reported that RNA transcription is triggered at sites of DNA damage where it fuels DDR activation. Mechanistically, exposed DNA ends generated at DNA double-strand breaks (DSBs) are recognized by the MRE11-RAD50-NBS1 (MRN) complex, which recruits RNA polymerase II to transcribe non-coding RNAs that promote the local retention of DDR factors at DSBs by favoring their RNA-dependent liquid-liquid phase separation (LLPS)^53–56^. Control of telomeric DDR is similarly dependent on transcripts generated upon telomere dysfunction. We reported that both strands of uncapped or dysfunctional telomeres are transcribed, resulting in C-rich and G-rich telomeric damage-induced long non-coding RNAs (dilncRNAs), called here teloC and teloG dilncRNAs, respectively^57,58^. Antisense oligonucleotides (ASO) are tools widely used to target RNAs in a sequence-specific manner, and many are in advanced clinical trials or are approved medicines^59^. ASO contain chemical modifications on their backbone and bases, which increase RNA binding strength and stability. We previously employed ASO with phosphorothioate backbones and locked-nucleic acid (LNA) base modifications in order to inhibit dilncRNAs^55,56^, including those induced at telomeres^57,58,60^. ASO-mediated inhibition of telomeric dilncRNAs results in diminished telomeric DNA damage signaling and repair while non-telomeric foci remain unaffected, demonstrating the effectiveness of ASO as telomere-specific DDR inhibitors in cultured cells and in animal models^57,58,60^.

Given the high level of replication stress, and thus damage, at ALT telomeres, here we studied the accumulation and potential role of telomeric dilncRNAs in the ALT pathway.

## Results

### ALT cells display elevated levels of telomeric dilncRNAs

**Fig. 1:**
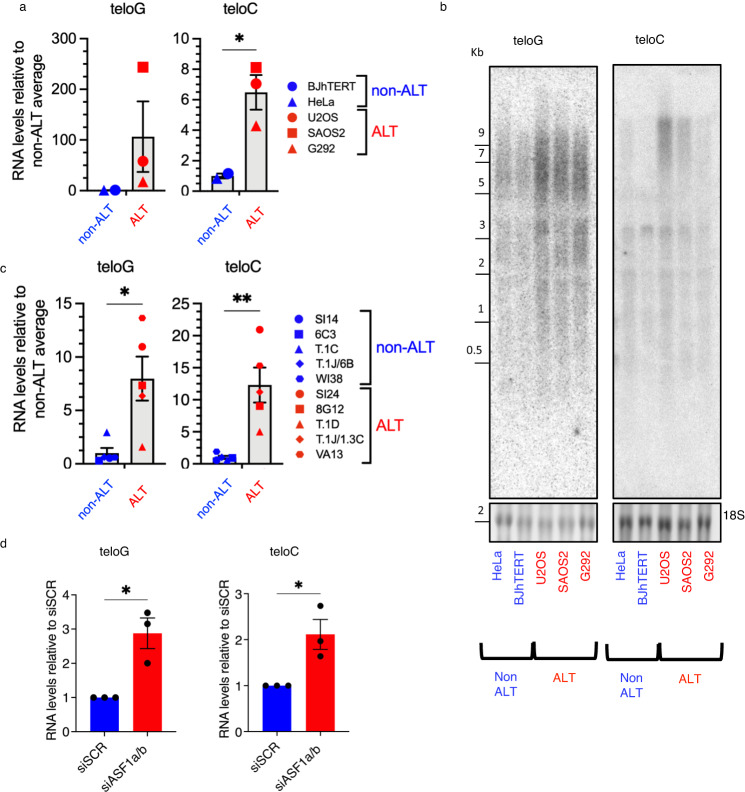
ALT cells display elevated levels of telomeric dilncRNAs. **a** Telomeric dilncRNAs were quantified by strand-specific RT-qPCR and normalized on RPLP0 gene; average values for each cell line were quantified in three biologically independent experiments; data are presented as mean values +/− SEM among *n* = 2 non-ALT and *n* = 3 ALT cell lines; two-tailed unpaired t-test, df = 3, *t* = 1.174 teloG, *t* = 3.718 teloC; *p = 0.0339. **b** Northern blot analysis of total RNA. Radiolabeled C-rich and G-rich telomeric oligonucleotides were used to probe for teloG and teloC dilncRNAs, respectively. EtBr-stained 18S RNA is shown as loading control; *n* = 2 biologically independent experiments, one representative image shown. **c** Telomeric dilncRNAs were quantified and reported as in (**a**); mean values for each cell line were quantified in *n* = 2 or 3 biologically independent experiments; data are presented as mean values +/− SEM among *n* = 5 non-ALT and ALT cell lines; two-tailed unpaired *t*-test, df = 8, *t* = 3.295 teloG, *t* = 4.127 teloC; *p = 0.0109, **p = 0.0033. **d** HeLa Long Telomere (LT) cells were transfected with siSCR (control) or siASF1a/b siRNAs twice per week, and telomeric dilncRNAs were quantified as in (**a**) 14 days after first siRNA transfection; data are presented as mean values +/− SEM, *n* = 3 biologically independent experiments; two-tailed unpaired *t*-test, df = 4, *t* = 4.208 teloG, *t* = 3.432 teloC; for teloG *p = 0.0136, for teloC *p = 0.0265. Source data are provided as a Source data file.

To test whether spontaneous telomeric DDR activation in ALT cells^8^ leads to telomeric dilncRNA accumulation, we compared telomeric dilncRNA levels between the ALT tumor cell lines U2OS, SAOS2 and G292, the non-tumor cell line BJhTERT and the telomerase-positive tumor cell line HeLa – the status of all ALT cell lines was confirmed by elevated C-circle levels (Supplementary Fig. 1a). Quantification of telomeric dilncRNAs by RT-qPCR and RNaseA-dependent northern blotting revealed elevated levels of both teloG and teloC species in ALT cells (Fig. 1a, b, Supplementary Fig. 1b).

Significant heterogeneity among ALT cells in their features such as C-circle levels^20,61^ and TIFs^8^ has been reported. To reduce the potential contributions of cell line discrepancies to telomeric dilncRNA levels, we examined a panel of paired ALT and non-ALT cell lines generated either through immortalization of normal cell lines or through fusions between telomerase-positive and ALT cells (Supplementary Table 1)—also here ALT status was confirmed by C-circle levels (Supplementary Fig. 1c). RT-qPCR analyses confirmed significantly higher levels of both teloG and teloC dilncRNAs in ALT cells with respect to paired non-ALT cells (Fig. 1c).

Next, to study the impact of ALT induction in a non-ALT cell line, thus in an isogenic setting, we measured telomeric dilncRNA levels following acute ALT activation upon ASF1a/b knockdown in HeLa Long Telomere (LT) cells^42^. HeLa LT cells were treated with siRNAs against ASF1a and ASF1b (siASF1a/b), or control siRNAs (siSCR), for two weeks. Knockdown efficiency was monitored by western blot, and ALT induction was confirmed by C-circle levels (Supplementary Fig. 1d, e). ALT induction was accompanied by an increase in both teloG and teloC species, as detected by RT-qPCR (Fig. 1d).

These results indicate that telomeric dilncRNA levels increase when cells activate ALT, prompting us to probe their potential functional role in this pathway.

### TeloC dilncRNAs are essential to maintain ALT cells viability

**Fig. 2:**
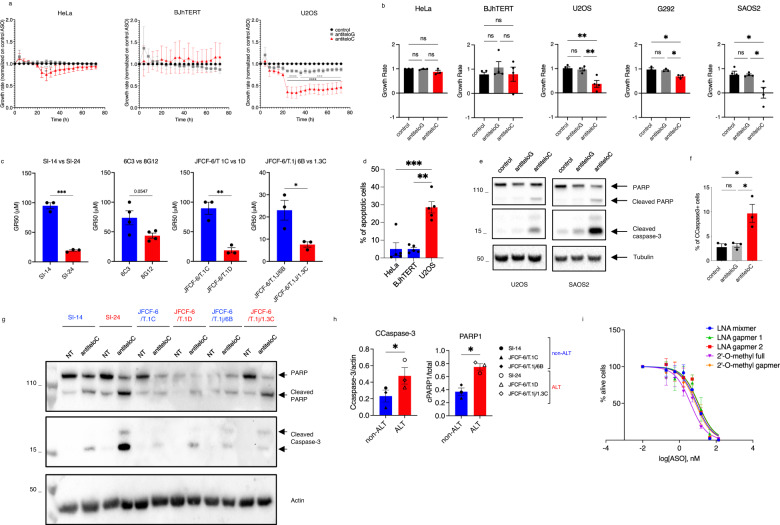
TeloC dilncRNAs are essential to maintain ALT cells viability. **a** Cells were transfected with 10 nM ASO, and growth rate was calculated by Incucyte; data are presented as mean values +/− SEM, *n* = 3 biologically independent experiments; two-way ANOVA, for HeLa control vs antiteloC 24 h *p = 0.0334, 28 h **p = 0.0063, 32 h *p = 0.0372; for HeLa antiteloG vs antiteloC 24 h *p = 0.0133, 28 h ***p = 0.0009, 32 h **p = 0.0092; for U2OS control vs antiteloC from 24 h to 72 h ****p < 0.0001; for U2OS antiteloG vs antiteloC from 24 h to 32 h ****p < 0.000, 36 h ***p = 0.003, 40 h ***p = 0.0002, 44 h ***p = 0.0007, 48 h ***p = 0.0004, from 52 h to 64 h ***p = 0.0002, 68 h ***p = 0.0003, 72 h ***p = 0.0005. **b** Cells were transfected with 20 nM ASO and growth rate was calculated three days later by resazurin; data are presented as mean values +/− SEM, *n* = 3 biologically independent experiments for HeLa (df = 8, *F* = 2.865) and G292 (df = 8, *F* = 8.454), *n* = 4 biologically independent experiments for BJhTERT (df = 11, *F* = 0.5318), U2OS (df = 11, *F* = 14.50) and SAOS2 (df = 11, *F* = 7.493); one-way ANOVA, ns = non-significant, for U2OS control vs antiteloC **p = 0.0024 antiteloG vs antiteloC **p = 0.0041; for G292 control vs antiteloC *p = 0.0224 antiteloG vs antiteloC *p = 0.0356; for SAOS2 control vs antiteloC **p = 0.0206 antiteloG vs antiteloC *p = 0.0212;. **c** Cells were treated with a range of concentrations of ASO antiteloC without transfection reagent and GR_50_ was calculated three days later with RealTime-Glo. When not determined, GR_50_ was underestimated to 100 μM (the highest concentration used in this experiment); data are presented as mean values +/− SEM, *n* = 3 biologically independent experiments for SI-14 and SI-24 (df = 4, *t* = 14.37), for JFCF-6/T1C and 1D (df = 4, *t* = 6.267), and for JFCF-6/T.1J6B and 1.3 C (df = 4, *t* = 3.349) and *n* = 4 biologically independent experiments for 6C3 and 8G12 (df = 6, *t* = 2.382); two-tailed unpaired *t*-test, for SI-14 vs SI-24 ***p = 0.0001; for JFCF-6/T1C and 1D **p = 0.0033; for JFCF-6/T.1J6B and 1.3 C *p = 0.0286 **d** Cells treated as in (**a**), and apoptotic cells counted by Incucyte three days after transfection; values reported as mean values +/- SEM, *n* = 5 biologically independent experiments; one-way ANOVA, df = 14, *F* = 34.71, HeLa vs U2OS ***p = 0.0004, BJhTERT vs U2OS **p = 0.0048. **e** Cells were transfected with 20 nM ASO and two days later analyzed by western blot; *n* = 2, one representative image shown. **f** U2OS cells were transfected with 20 nM ASO and fixed two days later for flow cytometry analysis; data are presented as mean values +/− SEM, *n* = 3 biologically independent experiments; one-way ANOVA, df = 8, *F* = 11.19; ns = non-significant, control vs antiteloC *p = 0.0135, antiteloG vs antiteloC *p = 0.0169. **g** Cells were treated with 20 μM of ASO antiteloC without transfection reagent and three days later analyzed by western blot. **h** The ratios between cleaved caspase 3 over actin and cleaved PARP1 over total PARP1 from the experiment shown in (**g**) were measured; data are presented as mean values +/− SEM, *n* = 3 non-ALT and *n* = 3 ALT cell lines; two-tailed paired *t*-test, df = 2, t(Ccaspase-3) = 4.458, t(PARP1) = 5.811,CCaspase3 *p = 0.0468, PARP1 *p = 0.0284. **i** U2OS cells were transfected with the indicated antiteloC ASO at a range of concentrations and live cells were counted by Incucyte three days after transfection; data are presented as mean values +/− SEM, *n* = 3 biologically independent experiments. Source data are provided as a Source data file.

Telomere maintenance in ALT cells requires telomeric DNA repair^15^. Targeting dilncRNAs with ASOs inhibits DDR signaling and DNA repair in a selective and sequence-dependent manner at genomic sites^55,56,62^, including at telomeres^57,58^. We thus tested the impact of ASO-mediated inhibition of telomeric dilncRNA in ALT cells. We designed ASOs complementary to either teloG or teloC dilncRNAs, called antiteloG and antiteloC ASO respectively, and control non-telomeric ASO, and determined their impact on HeLa, BJhTERT and U2OS cells, representing a telomerase-positive tumor cell line, a normal cell line, and an ALT tumor cell line, respectively. The growth rate (GR), rather than the relative number of cells, was calculated to compare drug sensitivities in multiple cell lines, since GR measurements account for differential division rates of cell lines, a factor that arbitrarily affects apparent drug response^63^. ASOs were transfected by lipofection and cell growth was monitored by IncuCyte^TM^ Live-Cell Analysis System continuously for three days. While HeLa and BJhTERT cells were unaffected by all ASOs tested, U2OS cells displayed a strong sensitivity to antiteloC ASO, but were unaffected by antiteloG and control ASO (Fig. 2a). Similar selective sensitivity was observed when ASOs were delivered without transfection (Supplementary Fig. 2a).

To study the impact of ASO treatments in other cell lines, we used resazurin to measure metabolically active cells before and after treatments. ALT tumor cells U2OS, SAOS2, and G292, and non-ALT cells HeLa and BJhTERT were transfected with ASOs and GRs were calculated three days later. GR measurements confirmed a quantitative and specific sensitivity of ALT cells to antiteloC ASO (Fig. 2b). ALT is most common among osteosarcomas, represented here by U2OS, SAOS2 and G292 cell lines. To extend our conclusions to glioblastomas (GBM), which are also very prone to activate ALT^7^, we compared relative sensitivities of well-characterized telomerase-positive TG16^64^ and ALT GBM14^65^ cell lines. We observed that while ALT GBM cells displayed a dose-dependent sensitivity to antiteloC ASO, telomerase-positive GBM cells were unaffected (Supplementary Fig. 2b). We further extended our analyses to matched pairs of ALT and non-ALT cell lines and calculated the respective antiteloC ASO GR_50_ values (the concentration at which antiteloC ASO reduce GR to 0.5). ALT cells consistently displayed lower GR_50_ values than telomerase-positive cells (Fig. 2c), indicative of increased sensitivity.

Since ASO antiteloC is a G-rich oligonucleotide, its effect could be explained by its potential G-quadruplex (G4)-forming capability, as G4-mediated cytotoxicity in cancer cells has been reported, even though this was unrelated to the telomere maintenance mechanisms^66^. In order to exclude the possibility that ASO antiteloC ALT-specific effect was dependent on G4s, we evaluated the growth rates of ALT and non-ALT cell lines treated with a published non telomeric G4-forming ASO^67^. The lack of response in both tested cell lines suggests that ALT cells are not specifically sensitive to G4-forming ASOs (Supplementary Fig. 2c).

The observed sensitivity to antiteloC ASO treatment could be caused by reduced cell proliferation or increased cell death, respectively known as cytostatic or cytotoxic effects. To distinguish between them, we sought for evidence of cell death by apoptosis. Live-cell imaging, western blot analysis for cleaved caspase-3 and PARP and flow cytometry-based detection of cleaved caspase-3 revealed induction of apoptosis following antiteloC ASO treatment relative to control ASO, resulting in significantly more apoptotic ALT than non-ALT cells (Fig. 2d–h, Supplementary Fig. 2d). Differently, when we probed for cellular senescence by senescence-associated β-galactosidase (β-gal) activity, it remained undetectable across all conditions tested (Supplementary Fig. 2e). Together, these results demonstrate a cytotoxic effect of antiteloC ASO treatment preferentially in ALT cells.

In order to broaden our conclusions, we tested the effects of ASO antiteloC treatment on a larger set of cells, including eight telomerase-positive cell lines and nine ALT cell lines, and calculated the respective antiteloC ASO GR_50_ values. ALT cells on average displayed significantly lower GR_50_ values than telomerase-positive cells (Supplementary Fig. 2f). To distinguish cytotoxic from cytostatic effects, a subset of cells was analyzed for caspase-3 activation, revealing apoptosis induction upon antiteloC ASO treatment preferentially in ALT cells (Supplementary Fig. 2g–h). In response to antiteloC ASO delivered without a transfection reagent, SAOS2 ALT cells displayed a low level of sensitivity (Supplementary Fig. 2f), but were significantly sensitive to antiteloC ASO delivered by transfection (Fig. 2b)—the difference could be explained by reduced unassisted/spontaneous ASO uptake in SAOS2, but alternative mechanisms associated with the specific genetic background of this cell line cannot be ruled out.

Some reports suggest the possibility that ALT and telomerase-mediated mechanisms of telomere maintenance may coexist in cancer cells^68^. We wondered if telomerase activity in U2OS cells may reduce sensitivity to teloC dilncRNA inhibition, and thus included U2OS cells expressing hTERT and hTERC (U2OS Telo) in the GR_50_ studies described above, which harbor high telomerase activity (Supplementary Fig. 2i). AntiteloC ASO sensitivity was similar between U2OS and U2OS Telo cells (Supplementary Fig. 2f), suggesting that the presence of telomerase activity does not mitigate ALT cell sensitivity to antiteloC ASO.

Different effects of antiteloC ASO on ALT and non-ALT were not due to different uptake rates in U2OS and HeLa cells as shown by internalization analysis experiments performed with a fluorescent-tagged ASO (Supplementary Fig. 2j).

Since ASO-mediated inhibition of teloC dilncRNAs results in cell death specifically in ALT cells, antiteloC ASO may represent a novel ALT-specific treatment. To investigate ASO efficacy in vivo, we developed a zebrafish-based xenograft model, a model organism widely used for its convenience^69,70^. ALT SAOS2 cells and ASOs were injected into the brain of two days post fertilization zebrafish larvae, and fish were collected for analyses one day later. Treatment with antiteloC ASO led to significantly more dead cells than control ASO (Supplementary Fig. 2k), demonstrating that antiteloC ASO are effective also in vivo to induce ALT cell death.

These results show that teloC dilncRNAs are essential to maintain viability and prevent apoptosis in a broad range of ALT cell lines.

### ALT sensitivity to teloC dilncRNA inhibition is independent from chemistry, design, and mechanism of action of the inhibitor

ASOs contain different chemical base modifications and can have different designs. The experiments described so far made use of LNA mixmers, ASOs bearing LNA modifications interspersed in the sequence, which inhibit target RNAs by steric hindrance. 2’-O-methyl modifications are common and effective alternatives to LNA. Gapmers, differently from mixmers, are ASOs with a central DNA region free from modifications and they inhibit target RNA through an RNaseH-mediated cleavage mechanism. To determine whether the effects observed were limited to a specific ASO chemistry or design, we compared ASOs all with the same sequence but with varying modifications, including mixmers with LNA, or 2’-O-methyl modifications (2’-O-methyl), or with a gapmer design containing LNA (Gapmer 2) or 2’-O-methyl (2’-O-methyl gapmer) modifications, as well as an additional gapmer LNA ASO with different sequences (Gapmer 1) (see Methods and Supplementary Table 2 for sequences). When directly compared, all different antiteloC ASOs had a similar impact on ALT cells viability (Fig. 2i) and comparable IC_50_ values, while all controls and antiteloG ASOs had little or no effect (Supplementary Fig. 2l, m).

RNA interference is a mechanism often employed to target RNA molecules. We therefore tested this approach to inhibit RNA functions and we designed siRNA targeting telomeric dilncRNA. We observed that antiteloC siRNA reduced U2OS ALT cells, but not HeLa non-ALT cells, growth rate relative to control siRNA (Supplementary Fig. 2n). Therefore, targeting teloC dilncRNA using multiple and unrelated tools, exploiting distinct mechanisms of action, leads to effective reduction of ALT cells growth in a specific manner.

Therefore, antiteloC ASO activity in U2OS ALT cells is sequence-dependent but chemistry- and design-independent and can be mirrored by RNA interference approaches.

### TeloC and teloG dilncRNAs have different roles in ALT cells

In our previous studies we observed equivalent roles for teloG and teloC dilncRNAs in controlling the DDR at telomeres^57,58^. However, ALT cells appear to be sensitive to the inhibition of teloC dilncRNAs only. We reasoned that TERRA, which shares the same G-rich telomeric sequence with teloG dilncRNAs and is highly expressed in ALT cells^29,71^, may compete for antiteloG ASO binding and thus reduce its impact. To test this, we compared ASO sensitivity in isogenic U2OS cell lines differing in TERRA expression levels as reported in ref. ^72^ and verified by us (Supplementary Fig. 2o). U2OS cells harboring reduced TERRA expression (U2OS-RTE) and control cells (U2OS-ctrl) were equally insensitive to antiteloG ASO (Supplementary Fig. 2p), suggesting that teloC and teloG dilncRNAs perform distinct functions at ALT telomeres.

### ALT cells rely on teloC dilncRNAs to cope with DNA replication stress

**Fig. 3:**
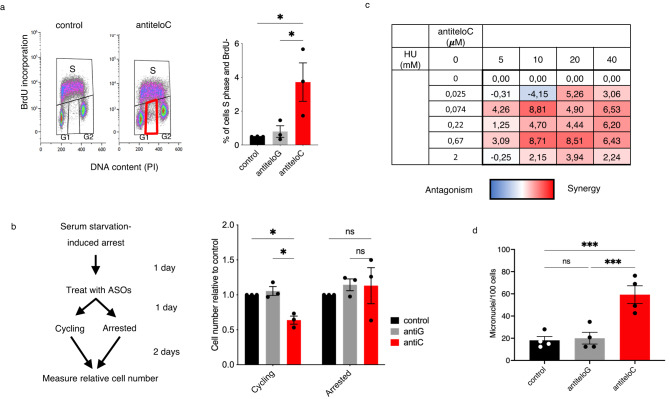
teloC dilncRNAs are required to maintain viability during replication stress. **a** U2OS cells were transfected with 20 nM ASO, and two days later pulsed with BrdU for 20 min and analyzed by flow cytometry; data are presented as mean values +/− SEM, *n* = 3 biologically independent experiments; one-way ANOVA, df = 8, *F* = 6.762, control vs antiteloC *p = 0.0279, antiteloG vs antiteloC *p = 0.0410; left, representative flow cytometry distribution; right, quantification. **b** Serum-starved SAOS2 cells were transfected with 20 nM ASO as indicated and relative cell number was quantified with resazurin; data are presented as mean values +/− SEM, *n* = 3 biologically independent experiments; one-way ANOVA, df = 8, *F*(cycling) = 21.04, *F*(arrested) = 0.26, ns = non-significant, control vs antic *p = 0.0444, antiG vs antic *p = 0.0235; left, experimental design; right, quantification. **c** U2OS cells were treated with a range of concentrations of HU and antiteloC ASO in absence of transfection reagent, and cell number was measured three days later by resazurin. Synergy was calculated according to the excess over Bliss additivism model (ref. ^77^). Lower Bliss values correspond to antagonism and are shown in blue, higher values correspond to synergy and are shown in red; data are presented as mean values, *n* = 3 biologically independent experiments. **d** U2OS cells were transfected with 20 nM ASO and, two days later, fixed, stained with DAPI, and micronuclei manually counted; data are presented as mean values +/− SEM, *n* = 4 biologically independent experiments; one-way ANOVA, df = 15, *F* = 18.10, ns = non-significant, control vs antiteloC ***p = 0.0005, antiteloG vs antiteloC ***p = 0.0008. Source data are provided as a Source data file.

Next, we investigated the functions of teloC dilncRNAs at U2OS ALT telomeres. ALT cells bear chronic telomeric DNA replication stress^23,26,73^, the response to which triggers telomere recombination and lengthening^74,75^ but, if exacerbated or the response interfered with, culminates in ALT-specific cell death^45,46^. To test the role of teloC dilncRNAs in these events, we analyzed the impact of their inhibition on cell-cycle phase distribution. We observed that teloC dilncRNA inhibition in U2OS cells caused an accumulation in S-phase (Supplementary Fig. 3a). When we also analyzed S-phase progression by including BrdU incorporation, we observed that antiteloC ASO treatment led to the appearance of a population of BrdU-negative S-phase cells (Fig. 3a), highlighting an S-phase DNA replication arrest. These results suggest that teloC dilncRNAs are necessary for ALT cells to efficiently progress through S-phase.

If ALT cells rely on teloC dilncRNAs to pass smoothly through S-phase, we reasoned that antiteloC ASO treatment effects could be DNA replication-dependent. To test this, we compared ASO sensitivity of cycling and non-cycling ALT cells. SAOS2 cells were arrested in G1 by serum-starvation (Supplementary Fig. 3b), transfected with ASOs, and then either kept arrested or allowed to resume cycling by serum supplementation (Fig. 3b, left). Under these conditions, we observed that, while cycling cells were sensitive to teloC dilncRNA inhibition, arrested cells were not (Fig. 3b, right), suggesting that ALT cell sensitivity to teloC dilncRNA inhibition is DNA replication-dependent.

Since DNA replication stress reduction by halted proliferation prevented sensitivity to teloC dilncRNAs inhibition in SAOS2, we tested if, inversely, increased replication stress may lead to greater sensitivity. We thus employed hydroxyurea (HU) to induce DNA replication stress by dNTP depletion and asked if ALT sensitivity to antiteloC ASO was affected. ALT tumor U2OS cells were treated with HU and antiteloC ASO at a range of concentrations, and cell viability was quantified three days later. Synergy between HU and antiteloC ASO was calculated according to the Bliss independence model—Bliss synergy values represent the percent of cells that die more than expected in response to a combination of drugs, and higher values suggest greater synergy^76,77^. Synergy between antiteloC ASO and HU in reducing cell viability was observed at a wide range of concentrations (Fig. 3c).

Unchecked replication stress leads to increased levels of micronuclei, which are a marker of genomic instability^78,79^. U2OS treated with antiteloC ASO displayed significantly higher levels of micronuclei compared to ASO antiteloG and control condition, while micronuclei formation in HeLa non-ALT cells was unchanged by telomeric ASO treatment (Fig. 3d, Supplementary Fig. 3c–e). This observation is consistent with a role for teloC dilncRNA in preventing rampant replication stress and genomic instability specifically in ALT cells.

Overall, these results suggest that teloC dilncRNA inhibition in ALT cells may impair an efficient response to endogenous replication stress, and this is a possible explanation for the sensitivity of ALT cells to antiteloC ASO.

### Inhibition of teloC dilncRNAs upregulates unproductive break-induced replication

The increased levels of micronuclei in ASO antiteloC-treated cells may indicate that teloC dilncRNA plays a role in ALT cells during telomeric recombination intermediate processing, as coordination of this process is necessary to prevent the formation of micronuclei^17,80^. Two opposite and competing recombination intermediate processing pathways have been proposed at ALT telomeres: resolution, mediated by the SLX4 complex, and dissolution, promoted by the BTR (BLM-TOP3A-RMI) complex, whose recruitment is dependent on PML^17,81^.

**Fig. 4:**
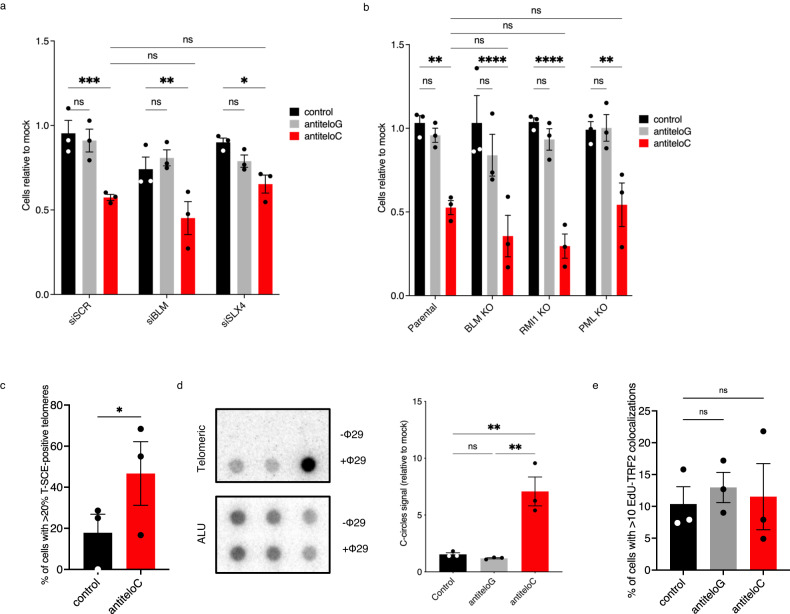
teloC dilncRNA inhibition upregulates unproductive break induced replication. **a** U2OS cells were transfected twice with the indicated siRNAs prior to transfection of siRNAs and 20 nM ASO, and cell viability was monitored three days later with resazurin; data are presented as mean values +/− SEM, *n* = 3 biologically independent experiments; two-way ANOVA, ns = non-significant, siSCR ***p = 0.0008, siBLM **p = 0.0084, siSLX4 *p = 0.0245. **b** Cells were transfected with 20 nM ASO and relative cell viability was measured three days later with resazurin; data are presented as mean values +/− SEM, *n* = 3 biologically independent experiments; two-way ANOVA, ns = non-significant, parental **p = 0.0017, BLM KO and RMi1 KO ****p < 0.0001, PML KO **p = 0.0051. **c**–**e** U2OS cells were transfected with 20 nM ASO as indicated and collected two days later for analysis. **c** Metaphases were subjected to COFISH and stained with PNA probes; data are presented as mean values +/− SEM, *n* = 3 biologically independent experiments; two-sided, paired *t* test, **p = 0.0499. **d** C-circle levels were analyzed by CCA, signals were normalized on ALU; values reported as the difference between +Φ29 and −Φ29, data are presented as mean values +/− SEM, *n* = 3 biologically independent experiments; one-way ANOVA, df = 8, *F* = 20.01, ns=non-significant, control vs antiteloC **p = 0.0044, antiteloG vs antiteloC **p = 0.0032. **e** U2OS were pulsed for 2 h with 10 μM EdU before fixation. Telomeric non-S DNA synthesis was quantified as colocalization of TRF2 and EdU foci in cells with less than 20 EdU foci; data are presented as mean values +/− SEM, *n* = 3 biologically independent experiments, one-way ANOVA, ns = non-significant. Source data are provided as a Source data file.

To test if teloC dilncRNA functions lie genetically within these pathways, we acutely knocked down BLM or SLX4 in U2OS and tested them for antiteloC ASO sensitivity. Knock down of BLM or SLX4 via siRNA resulted in similar sensitivities to antiteloC ASO as control siRNA-treated cells (Fig. 4a). In addition, we monitored cell viability following ASO treatment of U2OS cell lines that were individually knocked out for BLM, RMI1, or PML^81^. We observed that parental wild type and knockout cell lines displayed similar sensitivities to antiteloC ASO (Fig. 4b). Knockout and knockdown of BLM, RMI1, and PML were all confirmed in the samples tested and the opposite roles of SLX4 and BLM in ALT telomeric recombination processing was confirmed by C-circle analysis (Supplementary Fig. 4a–d). These results indicate that teloC dilncRNA activity in preserving U2OS ALT cells viability is independent from the recombination intermediates processing step of ALT.

The processing of recombination intermediates at ALT telomeres can be investigated by quantifying the products of the two alternative pathways. SLX4-mediated resolution leads to telomeric sister chromatid exchanges (T-SCE) and no net telomere extension, while BTR complex-mediated dissolution leads to non-S phase telomeric elongation and C-circle formation^17,82^. Treatment with antiteloC ASO increased both T-SCE and C-circle levels (Fig. 4c, d, Supplementary Fig. 4e), but had no effect on non-S telomeric DNA synthesis colocalizing with telomeres (Fig. 4e, Supplementary Fig. 4i) or on APBs (Supplementary Fig. 4f, i), suggesting an upregulation of non-productive ALT telomeric processing. We also observed that number of APBs per cell was reduced by treatment with antiteloC ASO (Supplementary Fig. 4g). Although APBs are sensitive to altered cell cycle phase distribution^83^, the observed APB number reduction was also associated with an increase in PML foci volume, suggesting that a distinct mechanism, based on clustering of APBs, may underly our observation (Supplementary Fig. 4h).

Finally, telomere fluorescence in situ hybridization (FISH) and terminal restriction fragment (TRF) assays could not reveal significant appreciable length or structural changes following antiteloC ASO treatment (Supplementary Fig. 4j, k) and Telomere Shortest Length Assay (TeSLA), which is exquisitely sensitive to telomere lengths changes^84^, also did not reveal an apparent impact on telomeres length in the cells tested (Supplementary Fig. 4l), suggesting that teloC dilncRNA inhibition boosts ALT engagement without affecting telomeric elongation.

### Inhibition of teloC dilncRNAs results in altered DDR factor recruitment to ALT telomeres

**Fig. 5:**
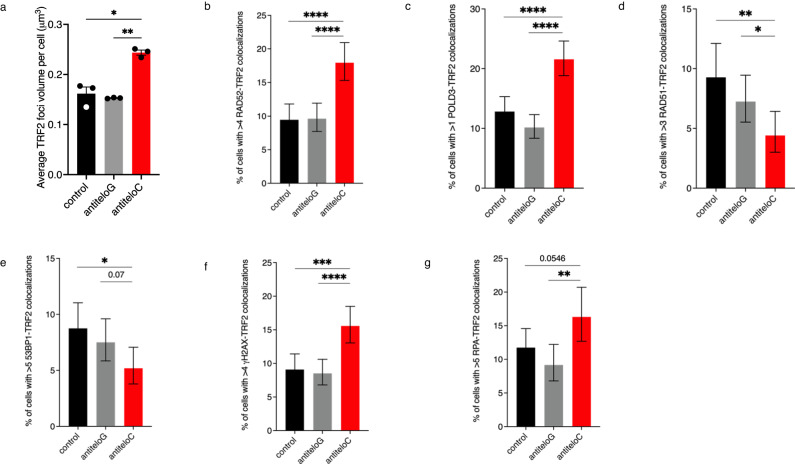
teloC dilncRNA inhibition alters the engagement of DDR factors at ALT telomeres. **a**–**g** U2OS cells were transfected with 20 nM ASO as indicated and collected two days later for analysis. **a** Average TRF2 foci size per cell was measured with an automated pipeline in ImageJ; data are presented as mean values +/− SEM, *n* = 3 biologically independent experiments; one-way ANOVA, *p = 0.049, **p = 0.0041. **b** Cells were immunostained for RAD52 and TRF2 and colocalizations were scored; data are presented as percentages +/− 95% confidence interval; *n* =  more than 710 cells examined over 3 biologically independent experiments; two-sided Fisher’s exact test, ****p < 0.0001. **c** Cells were immunostained for POLD3 and TRF2 and colocalizations were scored; data are presented as percentages +/− 95% confidence interval; *n* =  more than 680 cells examined over 3 biologically independent experiments; two-sided Fisher’s exact test, ****p < 0.0001. **d** Cells were immunostained for RAD51 and TRF2 and colocalizations were scored; data are presented as percentages +/− 95% confidence interval; *n* =  more than 500 cells examined over 3 biologically independent experiments; two-sided Fisher’s exact test, **p = 0.0021, *p = 0.0404. **e** Cells were immunostained for 53BP1 and TRF2 and colocalizations were scored; data are presented as percentages +/− 95% confidence interval; n= more than 710 cells examined over 3 biologically independent experiments; two-sided Fisher’s exact test, *p = 0.0093. **f** Cells were immunostained for *γ*H2AX and TRF2 and colocalizations were scored; data are presented as percentages +/− 95% confidence interval; *n* =  more than 680 cells examined over 3 independent biological replicates; two-sided Fisher’s exact test, ***p = 0.0002, ****p < 0.0001. **g** Cells were immunostained for RPA and TRF2 and colocalizations were scored; data are presented as percentages +/− 95% confidence interval; *n* =  more than 325 cells examined over 3 biologically independent experiments; two-sided Fisher’s exact test, **p = 0.0035. Source data are provided as a Source data file.

ALT telomeres are associated with chronic DDR activation^8^. We previously demonstrated roles of dilncRNAs in supporting telomeric DDR and repair^57,58^. Considering the increased levels of some BIR features upon antiteloC ASO treatment, such as C-circles and APBs clustering, we investigated if teloC dilncRNA might have a role in the recruitment of BIR and general DDR factors to ALT telomeres. To do so, we quantified by immunofluorescence the frequency of DDR factors colocalization with telomeres, marked by the telomeric marker TRF2. The observation of TRF2 foci alone provided information about telomeric DDR status: antiteloC treatment increased the volume of TRF2 foci, suggesting either telomeric clustering, or telomere chromatin decompaction, which are both possible indications of enhanced telomeric DDR activation (Fig. 5a, Supplementary Fig. 5a)^12,85^.

RAD52 and POLD3 are two key BIR proteins that promote ALT activity^11,15^. Telomeric recruitment of both of these factors was upregulated upon ASO antiteloC treatment, suggesting a role for teloC dilncRNA in restricting BIR engagement at ALT telomeres (Fig. 5b, c, Supplementary Fig. 5c, d, f, g). Increased localization of BIR factors to ALT telomeres could result from the deregulated recruitment of other DDR-related proteins. RAD51 and 53BP1 are two fundamental players in the response to DNA damage^86,87^ that localize to ALT telomeres^8,9,88^, and recruitment of both of them to DNA lesions is dependent on dilncRNAs^53,55,62^. Consistently, inhibition of teloC dilncRNA reduced RAD51 and 53BP1 recruitment to ALT telomeres (Fig. 5d, e, Supplementary Fig. 5b, c, h, i). RAD51 plays an important role in preserving ALT telomeres integrity^89^, while BIR is both the source and the outcome of replication stress^90^. ALT cells treated with antiteloC ASO displayed higher levels of both RPA and γH2AX at telomeres, suggesting elevated levels of telomeric replication stress and DNA damage (Fig. 5f, g, Supplementary Fig. 5d, e, j, k).

These results suggest a critical role of teloC dilncRNA in coordinating the engagement of selected DDR factors at ALT telomeres and prevent accumulation of telomeric damage.

## Discussion

Here we describe a novel role for teloC damage-induced long non-coding RNA (dilncRNA) in ALT tumor cells. We observed higher levels of telomeric dilncRNAs in ALT cells compared to telomerase-positive cancer cells and normal cells (Fig. 1a–d). TeloC dilncRNA plays important functions in the ALT pathway, as its inhibition by sequence-specific ASO, independent from chemistry and design, induces ALT-selective cell death (Fig. 2, Supplementary Fig. 2). We observed that teloC dilncRNA allows for regular progression through S-phase (Fig. 3a, Supplementary Fig. 3a), hence only proliferating, but not arrested, ALT cells are sensitive to teloC dilncRNA inhibition (Fig. 3b) and DNA replication stress induced by HU treatment exacerbated ALT cell sensitivity to inhibition of teloC dilncRNAs (Fig. 3c). Treatment with antiteloC ASO increased micronuclei formation (Fig. 3d), supporting the notion that these RNAs are necessary to cope with DNA replication stress, to maintain ALT cells genome integrity and prevent cell death by apoptosis (Fig. 2d, h). Differently, functions of teloC dilncRNA at telomeres in non-ALT cells, which exhibit less endogenous replication stress than ALT cells^24,91^, are dispensable for survival (Fig. 2a–d, g, h, Supplementary Fig. 2a, b, d, f–h).

A role for teloC dilncRNA in managing replication stress at ALT telomeres can be ascribed to its ability to recruit replication stress-relieving factors. RAD51 protects stalled DNA replication forks both by protecting ssDNA^92^ and by enacting fork reversal^93^. It localizes to damaged ALT telomeres and prevents telomeric DNA damage accumulation^12,24^. 53BP1 localizes to ALT telomeres, but its role is less studied^88^. It is mainly known for its functions in DSB repair, but a role in protection and restart of stalled replicative forks has also been invoked^94,95^. Inhibition of teloC dilncRNA decreased the recruitment of RAD51 and 53BP1 (Fig. 5d, e), thus reducing the contribution of these two factors in easing replication stress.

DNA replication stress, including at telomeres, is associated with stalled DNA replication forks which, if not promptly repaired by DDR factors to allow replication to restart, may collapse resulting in single-sided DSBs^43^. These can be repaired by BIR^44^, which elongates ALT telomeres^15^ and is associated with T-SCE and C-circles^17^. We observed that teloC dilncRNA inhibition promotes T-SCE and C-circle formation (Fig. 4c, d). Higher levels of C-circles could also reflect an enhancement in their stability, but the increased recruitment of BIR-related factors, such as RAD52 and POLD3 (Fig. 5b, c) points in the direction of increased C-circles production, consistent with a role of teloC dilncRNA in preventing fork collapse and BIR initiation. However, telomeric non-S synthesis was not fueled upon antiteloC ASO treatment, suggesting that, in absence of teloC dilncRNA, BIR engagement is apparently not productive (Fig. 4e, Supplementary Fig. 4f).

Accumulation of replication stress^96^ and, possibly, the engagement of unproductive BIR, may damage telomeric DNA to the point in which ALT cells succumb to apoptosis. We observed that, while telomeric fragility, fusion, loss, and length were apparently unaffected (Supplementary Fig. 4j–l), larger telomeric signals (Fig. 5a) and increased levels of RPA and γH2AX foci (Fig. 5f, g) appeared in interphase pointing to telomeric dysfunction and DNA damage accumulation^12,85^. The lack of structural telomeric abnormalities that we observed in metaphases may reflect sudden and catastrophic telomeric dysfunction following teloC dilncRNA inhibition, which could prevent cells with massively damaged telomeres from entering mitosis and being represented in metaphase spreads.

**Fig. 6:**
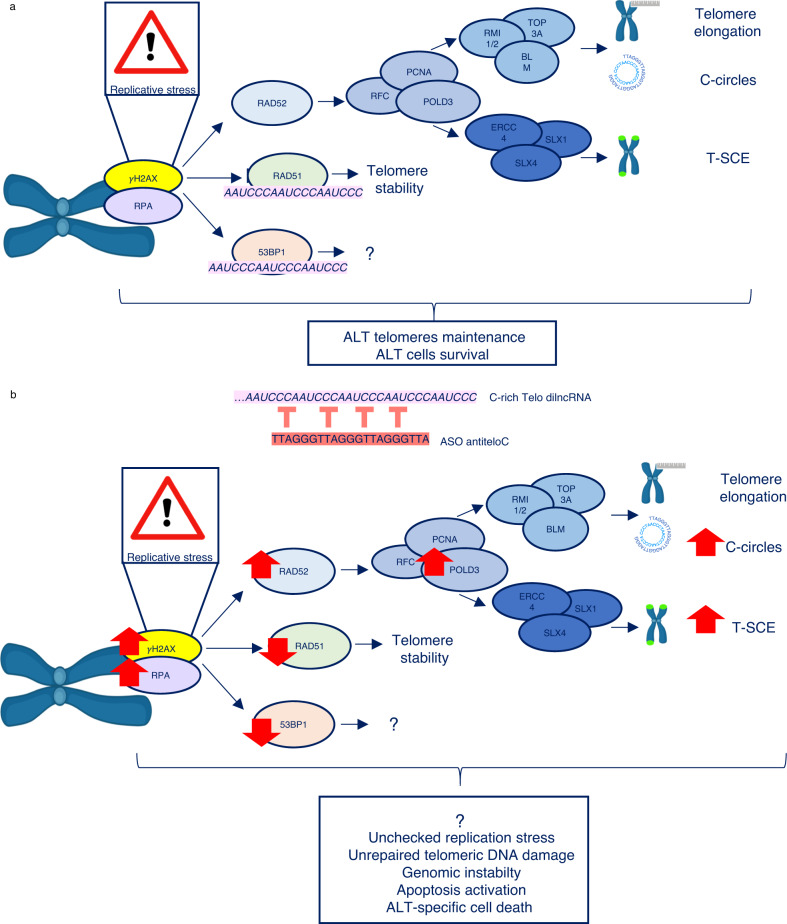
Model of proposed teloC dilncRNA function at ALT telomeres. **a** Proposed mechanism in unchallenged ALT cells. **b** Proposed mechanism in ASO antiteloC treated ALT cells. Created with BioRender.com.

Overall, our results are consistent with a model (Fig. 6) in which teloC dilncRNA plays a vital role in regulating the engagement of DDR factors at ALT telomeres. Unchallenged ALT cells deal with the high levels of telomeric replication stress and DNA damage by coordinating a fine balance of DDR factors, some of them dependent on teloC dilncRNA for their localization. When this balance is maintained, ALT cells can elongate their telomeres efficiently through BIR, while other mechanisms guarantee telomeric stability and cell viability (Fig. 6a). When the functions of teloC dilncRNA are inhibited, the balance is broken: RAD51 and 53BP1 recruitment to ALT telomeres is impeded, therefore preventing the execution of their repair functions. ALT cells try to cope with this imbalance by boosting BIR engagement, but this only exacerbates telomere replication stress and telomeric instability, as demonstrated by the accumulation of RPA and γH2AX. The high levels of unchecked replication stress and unrepaired telomeric damage translates to genomic instability, activation of apoptosis, and eventually ALT-specific cell death (Fig. 6b). Interestingly, a recent study reported an ALT-specific sensitivity to the receptor tyrosine kinase inhibitor ponatinib that, similarly to what we report here, is associated with upregulation of TIFs, c-circles and micronuclei, but no increase in non-S telomeric DNA synthesis^97^.

ASO are established drugs approved to treat patients by targeting RNA involved in a variety of pathologies^59^. Although with significant less affinity, ASOs could also bind to single-stranded DNA but this possibility is unlikely in our settings and its biological impact low, for a number of reasons. AntiteloC ASO used here display a higher affinity for RNA than for DNA, with melting temperatures of 89 °C and 76 °C, respectively. In addition, the evidence that other tools, which are commonly used to target RNA, like siRNA or RNaseH-activating gapmer ASOs, display a similar effect also points to the specificity of our approach. We routinely use telomeric ASOs in mice with shortened or damaged telomeres and we reported reduced DDR^57^, improved tissue homeostasis and organ functions^58,60^, and increased healthspan and lifespan^58^: overall beneficial results which cannot be reconciled with ASOs binding to DNA and inevitably causing DNA damage and cell dysfunction. In addition, U2OS ALT cells with mutated PML, BLM and RMI1 bear significantly less teloC ssDNA^81^ and all these mutant cells displayed sensitivities to antiteloC ASO comparable to wild-type control cells (Fig. 4b). Moreover, the observation that antiteloC ASO treatment increases telomeric recruitment of RPA suggests that antiteloC ASO does not anneal to teloC ssDNA, which is instead upregulated because of DNA replication stress, and exposed to RPA binding (Fig. 5g). Finally, although ALT cells are characterized by the presence of teloC ssDNA, they display high levels of teloG ssDNA as well^18,98^, thus if ASOs reduced ALT cells viability by binding to telomeric ssDNA, this should occur with both telomeric ASOs. Taken together, these arguments point to the effects of telomeric ASOs as the result of targeting RNA, not DNA.

Previously, we reported that teloC and teloG dilncRNAs play similar roles in controlling telomeric DDRs^57,58,60^, and that damage-induced RNAs from either strand of non-telomeric DSBs control the DDR^55^ to a similar extent; however only the inhibition of teloC dilncRNAs affected ALT telomeres and cell viability. We excluded the possibility that TERRA, being naturally elevated in ALT cells^29,71,99^, could “sponge” antiteloG ASOs and prevent them from inhibiting teloG dilncRNAs (Supplementary Fig. 2o, p), indicating that in ALT cells teloG and teloC dilncRNAs have different roles. This may be the result of the differences between DSBs and sites of replication stress. We have previously found that MRN recruits RNA polymerase II to initiate transcription from both exposed DNA ends at DSBs^55,56,100^, but at a reversed or collapsed fork a single DNA end is generated. Transcription from this single-sided DSB produces teloC dilncRNA which may have different biology or functions.

ALT telomeres rely on the aberrant recruitment of various proteins^26,47,73^, which may be mediated in a sequence-specific manner by teloC dilncRNAs. TeloG dilncRNAs would not participate in recruiting the same proteins due to their different sequence: intriguingly RNA modifications events, such as m5C modifications^101^, can control HDR and such modifications may occur only on teloC dilncRNAs. Finally, the specific requirement for teloC dilncRNA at ALT telomeres may reflect the preferential ALT elongation of telomeric lagging strand^102^. The abundant gaps in the C-rich strand DNA, possibly due to faulty Okazaki fragments ligation^18^, would generate stretches of teloG ssDNA, where teloC dilncRNA could anneal to and modulate ALT mechanisms.

Multiple approaches to specifically target ALT cancer cells hinge on increasing replication stress, such as FANCM depletion^26^ and disruption of FANCM-BTR complex association^45^. Different from these approaches, inhibition of teloC dilncRNAs hinders the response to endogenous sources of replication stress. Importantly, the replication stress-inducing approaches described above, or other replication stress-inducing pharmacological agents, may synergize with teloC dilncRNA inhibition similar to HU (Fig. 3c), paving the road for highly specific and effective combinatorial therapies.

Inhibition of teloC dilncRNAs resulted in cytotoxicity across many ALT cell lines, while non-ALT cells were largely resistant, including non-tumoral cell lines BJhTERT and RPEhTERT (Fig. 2, Supplementary Fig. 2). Recent years have seen numerous proposed ALT-specific treatments^75,103,104^, however the only method entailing DDR manipulation, pharmacological inhibition of ATR kinase activity by ATRi, remains controversial^49–52^. Thus, teloC dilncRNA inhibition is an ALT-specific treatment effective by selective DDR inhibition. As ASOs are drugs approved for various pathologies with some in advanced clinical trials for the treatment of cancer^59^, our approach has a promising translational potential for ALT cancer treatment.

## Methods

### Cell culture

HeLa, WI-38, and WI-38 VA13 (ATCC) were grown in MEM supplemented with 10% fetal bovine serum (FBS), 2 mM L-glutamine, 1% nonessential amino acids, and 1% sodium pyruvate. BJ-hTERT (ATCC) were grown in DMEM supplemented with 10% FBS, 2 mM L-glutamine, 20% M199 and kept in selection with 10 μg/ml hygromycin. U2OS (ATCC), HCT116 (DSMZ) and G-292 (ECACC) were grown in McCoy’s 5a supplemented with 10% FBS and 2 mM L-glutamine. For GR50 experiments in Supplementary Fig. 2f U2OS were grown in McCoy’s 5a supplemented with 15% FBS and 2 mM L-glutamine. SAOS2 (DSMZ) were grown in McCoy’s 5a supplemented with 15% FBS and 2 mM L-glutamine. IMR90 SW26 and SW39 were grown in DMEM supplemented with 10% FBS, 2 mM L-Glutamine, 20% M199. RPE hTERT (ATCC) were grown in DMEM/F12 supplemented with 10% FBS, 2 mM L-Glutamine, 15 mM HEPES, 0.5% sodium pyruvate. JFCF-6/T.1J/6B, JFCF-6/T.1J/1.3 C, JFCF-6/T.1C, and JFCF-6/T.1D were grown in MEM with Glutamax or with 2 mM L-glutamine, supplemented with 10% FBS, 1% sodium pyruvate, and 1% nonessential amino acids. SI14, SI24, 6C3, and 8G12 were grown in DMEM supplemented with 10% FBS, 2 mM L-Glutamine, 1% sodium pyruvate, and 1% nonessential amino acids. SJSA-1 (ATCC) were grown in RPMI supplemented with 10% FBS and 2 mM L-glutamine. SJ-GBM2 (COGcell) were grown in IMDM supplemented with 20% FBS, 4mM L-Glutamine, 1X ITS (5 μg /ml insulin, 5 μg/ml transferrin, 5 ng/ml selenous acid). SKLU-1 (ICLC ECACC) were grown in MEM supplemented with 10% FBS, 2 mM L-Glutamine, 1% nonessential amino acids and 1% sodium pyruvate. GBM-14 were grown in DMEM/F12 with Glutamax supplemented with 2% B-27, 5 μg/ml heparin, 20 ng/ml bFGF and 20 ng/ml EGF. NCI-H295R (ATCC) were grown in DMEM/F12 supplemented with 2.5% Nu-serum, 0.00625 mg/ml insulin, 6.25 ng/ml selenium, 1.25 mg/ml bovine serum albumin, 0.00535 mg/ml linoleic acid. U2OS hTERT and control were grown in DMEM supplemented with 10% FBS, 2 mM L-Glutamine and kept in selection with 0.8 μg/ml puromycin. U2OS RTE and control were grown in DMEM supplemented with 10% FBS and 2 mM L-Glutamine. U2OS parental and BLM, RMI1, and PML KO were grown in DMEM supplemented with 10% FBS and 2 mM L-glutamine. TG16 were grown in DMEM/F12 3:1 supplemented with 2 mM L-glutamine, B27 without vitamin A, 5 µg/mL heparin, 20 ng/mL EGF, 20 ng/mL FGF2. For zebrafish xenograft experiments in Supplementary Fig. 2k SAOS2 (ATCC) cells were grown in DMEM supplemented with 10% FBS.

### C-circle assay

C-circle assay was performed as described in ref. ^105^. C-circle amplification was carried out using 5–20 ng of DNA. The product of the amplification was blotted onto a Hybond-N+ membrane (GE Healthcare). For detection of telomeric repeats, either a ^32^P-labeled 800 bp excision fragment from the Sty11 plasmid or a ^32^P-end-labeled telomeric oligonucleotides with the sequence 5′-CCCTAACCCTAACCCTAACCC-3′ was used as a probe. Hybridization was performed overnight at 65 °C in Church buffer (0.5 M sodium phosphate buffer pH 7.2, 1 mM EDTA pH 8.0, 7% SDS, 1% BSA) or at 37 °C in hybridization buffer (1.5X SSPE, 10% polyethylene glycol 8000, 7% SDS) respectively. Membranes were exposed to a phosphorimager screen, and subsequently imaged on a Typhoon Imager (GE Healthcare).

### RNA isolation

Total RNA from cultured cells was extracted with the Maxwell RSC simplyRNA Tissue Kit (Promega) according to the manufacturer’s instructions.

### Strand-specific real-time quantitative PCR

Detection of dilncRNAs was performed as previously described^57^, with some modifications. RNA samples were treated with TURBODNAse (Thermo Scientific) at 37 °C for 1 h. Total RNA was reverse transcribed using the Superscript III reverse transcriptase (Invitrogen) with strand-specific primers. cDNA was purified on a MicroSpin G-50 columns (Cytiva). qPCR was performed using SYBR Green I Master Mix (Roche) with 20 ng of cDNA, using RPLP0 for normalization. Each reaction was performed in triplicate. Primer sequences are reported below (5’→ 3’):

RPLP0: TTCATTGTGGGAGCAGAC; CAGCAGTTTCTCCAGAGC

Telomeric RNA reverse transcription: CCCTAACCCTAACCCTAA or GGGTTAGGGTTAGGGTTA Telomeric repeats: CGGTTTGTTTGGGTTTGGGTTTGGGTTTGGGTTTGGGTT; GGCTTGCCTTACCCTTACCCTTACCC TTACCCTTACCCT.

### Real-time quantitative PCR

RNA samples were reverse transcribed with the SuperScript VILO cDNA Synthesis Kit (Invitrogen). RT-qPCR was performed using SYBR Green I Master Mix (Roche). Rplp0 was used as a control gene for normalization. Each reaction was performed in triplicate.

Primer sequences are reported below (5’→ 3’):

Rplp0: TTCATTGTGGGAGCAGAC; CAGCAGTTTCTCCAGAGC

20q-1: CTGGTGCCAGAGTGGATT; CACCTGTTCTCTTTGTCTGG

20q-2: ACATGGGCGATACTCAGG; CCCACTACTGTGCCTCAA

20q-3: GAAGTTGCTGGGTTCTATGG; ATGGTGCAGACACTGTGG

### Northern blot

Northern blot was performed as described in ref. ^106^. Briefly, 10 μg of RNA were either mock treated, treated with 4 U of TURBODNAse (Thermo Scientific) or with 10 μg RNAseA (Qiagen). Samples were run in 1.2% agarose formaldehyde gels. After the run, the gel was treated with 50 mM NaOH for 20 min, washed and transferred overnight by capillary action in 20X SSC (Thermofisher) onto a Hybond NX neutral nylon membrane (GE Healthcare). The membrane was crosslinked twice with 1200 J of 254 nm UV light. Hybridization with radiolabeled 5’-(GGGTTA)_5_-3’ and 5’-(CCCTAA)_5_-3’ oligonucleotides was performed overnight at 37 °C in PerfectHyb Plus hybridization buffer (Sigma-Aldrich). The membranes were washed, exposed to a phosphorimager screen, and subsequently imaged on a Typhoon Imager (GE Healthcare).

### ASO sequences

Antisense oligonucleotides (ASOs) with a fully phosphorothioate backbone were produced by Exiqon (LNA) or IDT (2’-O-methyl). Sequences are reported in Supplementary Table 2.

ASOs were used at the indicated concentrations for transfection with Lipofectamine RNAiMAX (Invitrogen) or naked delivery in cultured cells.

### Telomerase repeat amplification protocol (TRAP) assay

TRAP assay was performed with the TRAPEZE® Merck-Millipore kit according to the manufacturer’s protocols.

### siRNA transfection

Transfections were carried out with Lipofectamine RNAiMAX (Invitrogen) according to the manufacturer’s instructions.

ON-TARGETplus SMARTpool Human ASF1A (L-020222-02-0005) and ASF1B (L-020553-00-0005) siRNAs were used at a final concentration of 0.4 nM or 0.8 nM, and siCTRL (D-001810-10-20) was used at a final concentration of 0.8 nM or 1.6 nM, respectively. Cells were transfected with siRNAs twice a week for the duration of the experiment.

ON-TARGETplus antiteloC siRNA (AGGGUUAGGGUUAGGGUUAUU, Dharmacon) were used at the concentrations indicated in the figures.

ON-TARGETplus SMARTpool short interfering RNA (siRNA) oligonucleotides (siCTRL D-001810-10-20; siBLM L-007287-00; siSLX4 L-014895-00-0005 Dharmacon) were used at a final concentration of 1.25 nM and transfection was repeated 3 times over 6 days.

### Immunoblot

Cells were lysed in Laemmli sample buffer (2% SDS, 10% glycerol, 60 mM Tris-HCl pH 6.8). 18–25 μg of whole cell extracts were resolved by SDS–polyacrylamide gel electrophoresis. Proteins were transferred to a nitrocellulose membrane, which was blocked in 5% milk in TBS-T. Membranes were incubated with the primary antibody for 3 h or overnight at 4 °C, washed, and incubated with a horseradish peroxidase-conjugated secondary antibody for 1 h at room temperature. Membranes were developed using SuperSignal West Pico PLUS (ThermoFisher) and acquired at a Chemidoc Imager (Biorad).

### Incucyte live-cell analyses

Cells plated in 96 MW and treated as indicated were monitored overtime using Incucyte S3 or S5 live-cell analysis system (Sartorius). For detection of apoptotic cells, Incucyte caspase 3/7 green dye for apoptosis (Sartorius) was added 1:1000 to cells one day after ASO transfection, to prevent interaction between ASO and dye. Growth rate (GR) was calculated as reported in ref. ^63^. For IC_50_ calculation, values were computed using Prism software.

### Cell viability assays

Resazurin: cell viability was assessed using In vitro toxicology assay kit, resazurin based (Sigma-Aldrich) in a 1:10 concentration. Fluorescence was measured 1 h or 2 h after addition of the reagent using the EnVision plate reader (PerkinElmer). The growth of the cells was calculated as the ratio between the fluorescence detected at the end of the experiment and the fluorescence detected at the time zero, taking in account the passage of the cells. Growth rate (GR) was calculated as reported in ref. ^63^.

GR50: 375-1000 cells were seeded one day prior to ASO treatment. Cell proliferation was monitored by RealTime-Glo (RTGlo) MT Cell Viability Assay (Promega). ASO and RTGlo reagent remained in the medium for the entire duration of the experiment and every 24 h RTGlo levels were measured. GR_50_ values were calculated using Prism software.

For HU treatments, 750–2000 cells were plated one day prior to HU treatment. ASO and HU were administered simultaneously, and cell viability was measured three days later using resazurin. Synergy was calculated according to Borisy et al.^76^.

### Senescence-associated-β-galactosidase assay (SA-β-Gal)

Cells were washed in PBS, fixed for 10 min in 4% PFA, washed, and incubated over night at 37 °C (in the absence of carbon dioxide) with fresh SA-β-Gal stain solution (pH 6.0): Potassium ferricyanide 5 mM, Potassium ferrocyanide 5 mM, Sodium dihydrogen phosphate 0.4 M, Sodium hydrogen phosphate 92 mM, Sodium chloride 150 mM, Magnesium dichloride 2 mM, and 5-bromo-4-chloro-3-indolyl-β-D-galactopyranoside 1 mg ml^−1^. Then, they were washed in PBS, fixed again in 4% PFA, permeabilized and stained with DAPI.

### Flow cytometry

Caspase-3 cleavage analysis: cells were fixed in 1% formaldehyde, washed, then fixed again in 75% ethanol at 4 °C. Fixed cells were permeabilized in 0.1% TritonX-100, blocked with 10% goat serum and stained for cleaved caspase 3 prior to analysis.

Cell cycle analysis: cells were fixed in 75% ethanol and incubated with PI (Sigma, 50 μg/ml) and RNAseA (Sigma, 250 μg/ml) overnight prior to analysis.

BrdU and cell cycle analysis: cells were pulsed with 33 μM BrdU (Sigma) for 20 min, collected by trypsinization, fixed in 75% ethanol, denatured in 2 N HCl at RT for 25 minutes, to which 3 ml of 0.1 M Sodium Borate was added for 2 min at RT, stained with anti-BrdU antibody, then incubated with PI (Sigma, 2.5 μg/ml) and RNAseA (Sigma, 250 μg/ml) overnight prior to analysis.

Samples were analyzed on a BD Facs CantoII. Analysis was done using ModfitLT 3.0 software.

### Immunofluorescence

Cells were fixed with 4% PFA. After incubation with blocking solution (0.5% BSA, 0.2% cold water fish gelatin in PBS 1X), cells were stained with primary antibodies for 1 h at room temperature, washed and incubated with secondary antibodies for 40 min at room temperature. Nuclei were stained with 1 μg/ml 4’-6-Diamidino-2-phenylindole (DAPI, Sigma-Aldrich). Samples were mounted in mowiol (Calbiochem) and imaged using a widefield Olympus Biosystems BX71 microscope.

For γH2AX, POLD3, RAD52, and 53BP1 staining covers were permeabilized in CSK buffer (10 mM PIPES [pH 7.0], 100 mM NaCl, 300 mM sucrose, 3 mM MgCl2, 0.7% Triton X-100) for 10 min at 4 °C prior to fixation.

For colocalization analysis, 3D stacks were acquired with a Leica SP8 confocal microscope and colocalizations events on images were identified by a software-based analysis (Arivis Vision4D, v3.1.4) using a custom pipeline. Foci were recognized using the Blob Finder method on images enhanced using the Particle Enhancement Filter 3D. Two foci were considered colocalizing if their boundaries were touching.

For telomeric non-S synthesis analysis, only cells with less than 20 foci of EdU were considered in the count, so to remove from the analysis S-phase cells.

Threshold number of colocalizations for statistical analysis was determined as the closest integer to the average value of colocalizations in control condition +1 SD.

### Chromosome orientation fluorescence in situ hybridization (COFISH)

Cells were labeled with 7.5 μM BrdU and 2.5 μM BrdC for 20 h prior to harvesting. After removal of nucleotide analogues, cells were incubated in 0.2 μg/ml colcemid for 3 h to induce an accumulation of cells in mitosis. Cells were harvested by trypsinization, swelled in 0.03 M sodium citrate and fixed in 3:1 methanol:acetic acid. Metaphase chromosomes were dropped onto glass slides, air-dried overnight and treated with 0.5 mg/ml RNAseA (Sigma). Slides were then washed, incubated with 5 μg/ml Hoechst 33258 (Invitrogen) for 15 min in the dark and exposed to 365 nm UV light. The nicked BrdU- and BrdC-containing DNA strands were digested with 3000 U/ml Exonuclease III (NEB). Next, slides were fixed in 4% formaldehyde for 2 min and incubated in 200 ml water, 200 mg pepsin (Sigma) and 168 μL 37% HCl for 10 minutes at 37 °C. Slides were then washed, fixed again in 4% formaldehyde for 2 min, dehydrated by ethanol series and air-dried. Dried slides were then hybridized with the Cy3-OO-(CCCTAA)_3_ telomeric PNA probe (Panagene) for G-rich DNA in hybridizing solution (70% formamide, 0.25% blocking reagent [Roche], 10 mM TrisHCl pH 7.2, 2.14 mM MgCl, 0.77 mM citric acid, 7.02 mM Na_2_HPO_4_) for 2 hr at RT. After hybridization, slides were rinsed with solution 1 (70% formamide, 10 mM Tris-HCl pH 7.2, 0.1% BSA) and with solution 2 (1× TBS, 0.08% Tween-20) then dehydrated in ethanol as above. Air-dried slides were incubated as above with an Alexa647-OO- [TTAGGG]_3_ PNA probe (PNABio). Slides were rinsed in solution 1 and 2, stained with DAPI, dehydrated in an ethanol series, air-dried and then mounted with mowiol or ProLong™ Diamond Antifade Mountant. Images were obtained with an Upright Olympus AX70 or Zeiss LSM 880 microscope. Only one probe was considered for analysis per treatment, as described in the text. Ends of chromosome that have at least one signal were counted as signal positive, and the amount of exchanges was estimated by the amount of chromosome ends with two signals. Slides were scored blind, and outliers were removed using the ROUT method in prism.

### Fluorescence in situ hybridization (FISH)

Cells were incubated in media containing 0.2 μg/ml colcemid for 3 h to induce an accumulation of cells in mitosis. Metaphase spreads were prepared as described for COFISH. Covers were denatured on a humid heat block at 80 °C for 1 min, then air-dried overnight in the dark. Slides were rehydrated, treated with 0.5 mg/ml RNAseA (Sigma), then washed and dehydrated in an ethanol series. Dried slides were incubated at 80 °C with the hybridization solution (see COFISH) and Alexa647- OO-[TTAGGG]_3_ PNA probe (PNABio, F1014) for 5 min, then hybridized at RT in a dark humid chamber for 2 h. Slides were rinsed in solution 1 and 2 (see COFISH), stained with DAPI, dehydrated in an ethanol series, air-dried and then mounted with mowiol or ProLong™ Diamond Antifade Mountant. Images were obtained with an Upright Olympus AX70 or Zeiss LSM 880 microscope. Fragile telomeres were scored if the telomeric signal was elongated, or appeared twice on one end, fused telomeres were scored if there was a telomeric signal between the ends of two chromosomes, and telomere loss was scored at a chromosome end with absent telomere signal.

### Terminal restriction fragment (TRF) assay

Cells embedded in agarose plugs were digested with 1 mg/ml proteinase K (Sigma) overnight. Plugs were incubated overnight at 37 °C with 50 U MboI and 50 U Alu1 (NEB) and loaded into a 1% agarose-0.5 × TBE gel. The gel was run for 24 h on a pulsed-field apparatus with the following settings: 6 V/cm, run time 24 h, angle 120°, initial switch time 5 s, final switch time 5 sec at 14 °C. The gel was stained with ethidium bromide and imaged, then depurinated in 0.25 M HCl for 30 min, denatured in 1.5 M NaCl 0.5 M NaOH for 30 min twice, and neutralized in 3 M NaCl, 0.5 M Tris- HCl pH 7.0 twice. The gel was transferred to Hybond N+ positively charged nylon membrane (GE Healthcare) by capillary action in 20X SSC, then crosslinked with 1200 J of 254 nm UV light. The membrane was pre-hybridized with Church buffer and hybridized overnight with a ^32^P -labeled 800 bp excision fragment from the Sty11 plasmid (see above) in Church buffer. The next day, the membrane was washed with pre-warmed Church wash (40 mM sodium phosphate buffer pH 7.2, 1 mM EDTA pH 8.0, 1% SDS) three times at 65 °C. The membrane was exposed to a phosphorimager screen, which was then developed on a Typhoon imager (GE Healthcare).

### Telomere shortest length assay (TeSLA)

TeSLA measurements were performed as described in Lai et al. (2017)^84^. Briefly, 50 ng of genomic DNA were mixed with 2 mM ATP, 0.5 μl T4 ligase (NEB), and Telo1-6 ligation oligos at 10 nM each in CutSmart buffer 1X (NEB), and incubated overnight at 35 °C. The mixture was digested with CviAII, MseI, NdeI and BfaI (NEB) to generate DNA fragments with 5′ AT and TA overhangs. The 5’ phosphate of these DNA fragments was removed using the shrimp alkaline phosphatase (rSAP, NEB). The DNA mixture was then incubated overnight at 16 °C with T4 DNA ligase, 1 mM ATP, 1 μM of AT adapter, and 1 μM of TA adapter in 1× CutSmart buffer.

Multiple PCRs were performed using FailSafe Enzyme Mix (Lucigen) with 1× FailSafe buffer H containing 0.25 μM AP/TeSLA-TP primers and 40 pg of ligated DNA. PCR products were loaded on a 0.85% agarose gel and run for 19 h at 1.5 V/cm. After gel electrophoresis, the amplified telomeres were detected by Southern blot.

### Antibodies

Actin (1:2000, A2228, Sigma-Aldrich), ASF1a (1:1000, 2990, Cell Signaling), ASF1b (1:1000, MA5-14836, Thermofisher), BLM (1:2000, ab476, Abcam), BrdU (1:5, 347580, BD Biosciences) cleaved Caspase-3 (9661, Cell Signaling, 1:50 for FACS, 1:1000 for immunoblot), γH2AX (1:1000, 9718, Cell Signaling), PARP1 (1:1000, Serotec), PML (1:100, SC966, Santa Cruz), POLD3 (1:500, H00010714-M01, Abnova) RAD51 (1:400, Ab213-100, Abcam), RAD52 (1:200, SC365341, Santa Cruz), RMI1 (1:200, NB100-1720, Novus Biologicals), SLX4 (1:1000, A302-270A, Bethyl), TRF2 (1:500, N20, Santa Cruz or 1:500, NB-11057130, Novus Biologicals), Tubulin (1:2000, T5168, Sigma), 53BP1 (1:1000, A300-272A, Bethyl).

### Animals and treatments

Zebrafish (wild-type strains AB and Tubingen) were maintained as described in (Westerfield, M.; Zon, L.I.; Detrich,W., III. Essential Zebrafish Methods: Cell and Developmental Biology; Academic Press: Oxford, UK, 2009). All animal experiments were concluded before 5 days post fertilization (dpf) and performed in accordance with European guidelines and regulations, according to which no ethical approval is required for zebrafish larvae up to 5 dpf. Zebrafish were incubated at 28.5 °C in E3 water till 24 h post fertilization and then at 33 °C, in preparation for the transplantation of human cells. SAOS2 cells were harvested at 70% confluence and resuspended in sterile PBS at a concentration of 50,000 cells/μL. Live-cell labeling of the nucleus was performed with Hoechst 33342, diluted 1:10,000, before transplantation into the zebrafish larvae, to allow visualization of the cells after implantation. Approximately 5–10 nL containing 50–100 cells were transplanted in the hindbrain of 2 days post fertilization (dpf) zebrafish. Fish were returned to the incubator, incubated at 33 °C and fixed one day post-transplantation. Transplanted zebrafish embryos were injected within 1–2 h post-transplantation with 0.5–1 ng LAC (control) or antiteloC ASO (sequences in Supplementary Table 2) diluted in Danieau buffer. Transplanted cells were imaged in fixed larvae with a confocal microscope (Leica, SP5), using ×10 and ×40 objective and optical sectioning, followed by 3D reconstruction and manual cell counting using ImageJ. Transplanted cells have nuclei labeled by Hoechst 33342, and apoptotic bodies in death cells are easily recognizable by chromatin compaction and DNA fragmentation stained by Hoechst 33342^107^.

### Statistical analysis

Values shown represent mean ± standard error of the mean (SEM) or standard deviation (SD), or as percent ± 95% confidence interval as indicated in figure legends. *P* values were calculated by the indicated statistical tests, using Prism software. In figure legends, n indicates the number of independent experiments or technical replicates, as indicated.

### Reporting summary

Further information on research design is available in the Nature Portfolio Reporting Summary linked to this article.

### Supplementary information

Supplementary Information

Reporting Summary

### Source data

Source Data

## Data Availability

All data supporting the findings of this study are available within the paper and its Supplementary Information. Source data are provided as a Source Data file. Source data are provided with this paper.
