# Supplementary material for: Alternative lengthening of telomeres (ALT) cells viability is dependent on C-rich telomeric RNAs
Source: Nat Commun. 2023 Nov 4;14:7086. doi: 10.1038/s41467-023-42831-0 (PMC10625592; doi:10.1038/s41467-023-42831-0)
Supplement: Supplementary file 1 — Supplementary Information [file 41467_2023_42831_MOESM1_ESM.pdf]

Cell line	TMM	Parental cell line	Method of immortalization	Reference
SI14	Telomerase	SW39 (telomerase-positive) and IMRB (ALT)	Fusion between SW39 and IMRB (both SV40 Large T-antigen immortalized from IMR90)	Episkopou 2014
SI24	ALT			
6C3	Telomerase			Episkopou 2019
8G12	ALT			
JFCF-6/T.1C	Telomerase	JFCF-6 (mortal)	SV40 Large-T antigen immortalized	Yeager 1999
JFCF-6/T.1D	ALT			Lovejoy 2012
JFCF-6/T.1J/6B	Telomerase			Jiang 2009
JFCF-6/T.1J/1.3C	ALT			Lovejoy 2012
WI38	-	WI38 (mortal)	-	Bryan 1997
VA13	ALT		SV40Large T-antigen immortalized	

Supplementary table 1: Additional information on paired ALT and non-ALT cell lines.

	Control	antiteloG	antiteloC
LNA mixmer	antiLac TTATCCGCTCACAATTCCACAT;<br>RB4 TCGGGGTAGCGGCTGAAGCA;<br>G4-forming ASO GGTTGGTGTGGTTGG	CCCTAACCCTAACCCTAACCC	GGGTTAGGGTTAGGGTTAGGG
LNA gapmer1	AACACGTCTATACGC	TAACCCTAACCCTAAC	TTAGGGTTAGGGTTAG
LNA gapmer 2	TAACACGTCTATACGCCCA	CCCTAACCCTAACCCTAACCC	GGGTTAGGGTTAGGGTTAGGG
2’-O-methyl full	UUAUCCGCUCACAAUUCCACAU	CCCUAACCCUAACCCUAACCC	GGGUUAGGGUUAGGGUUAGGG
2’-O-methyl gapmer	UUAUCCGCUCACAAUUCCACAU	CCCUAACCCUAACCCUAACCC	GGGUUAGGGUUAGGGUUAGGG

Supplementary table 2: Sequences of the ASO used in this study.

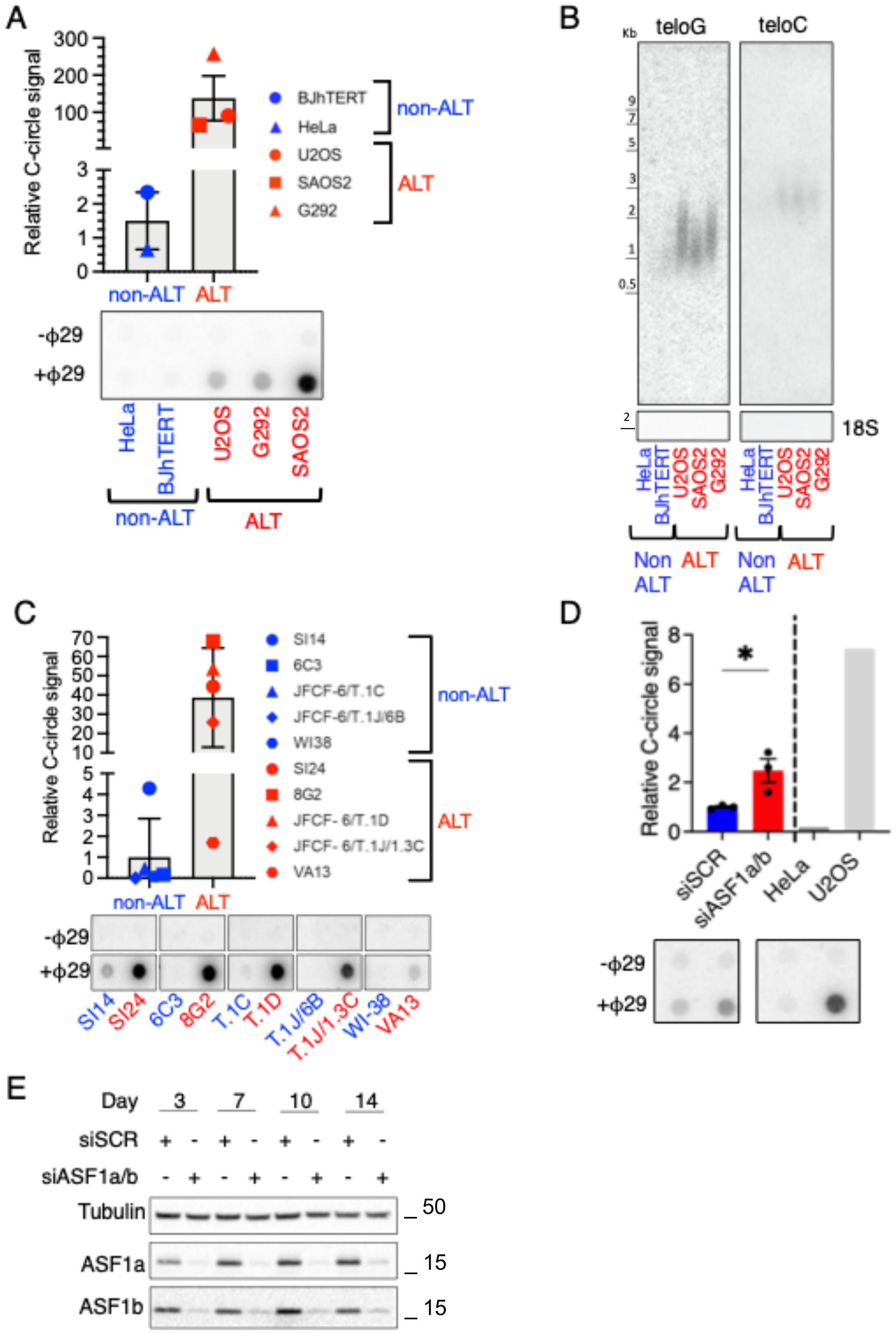
A
Relative C-circle signal
non-ALT
ALT
BJhTERT
HeLa
U2OS
SAOS2
G292
-ϕ29
+ϕ29
B
teloG
teloC
Kb
18S
Non ALT
ALT
C
SI14
6C3
JFCF-6/T.1C
JFCF-6/T.1J/6B
WI38
SI24
8G2
JFCF- 6/T.1D
JFCF- 6/T.1J/1.3C
VA13
D
siSCR
siASF1a/b
E
Day
Tubulin
ASF1a
ASF1b

Supplementary figure 1: ALT cells characterization. A C-circle levels were analyzed by C-circle assays (CCA). Values were normalized on input DNA. CCA for each cell line was performed once; data are presented as mean values +/- SEM, n=2 non-ALT cell lines and n=3 ALT cell lines. **B** Northern blot analysis of total RNA digested with RNAseA as a negative control. Radiolabeled C-rich and G-rich telomeric oligonucleotides were used to probe for teloG and teloC dilncRNAs, respectively; n=2 biologically independent experiments, one representative image shown. **C** C-circle levels analyzed as in A. CCA for each cell line was performed once; data are presented as mean values +/- SEM, n=5 non-ALT and n=3 ALT cell lines**. D** samples from fig 1D were analyzed for C-circle levels as in A, one representative image shown; data are presented as mean values +/- SEM, n=3 biologically independent experiments; HeLa and U2OS n=1; two-tailed unpaired t-test, df=4, t=3.113, *p=0.0358. **E** samples from fig 1D were analyzed for ASF1a/b protein levels by western blot; n=3 biologically independent experiments, one representative image shown.

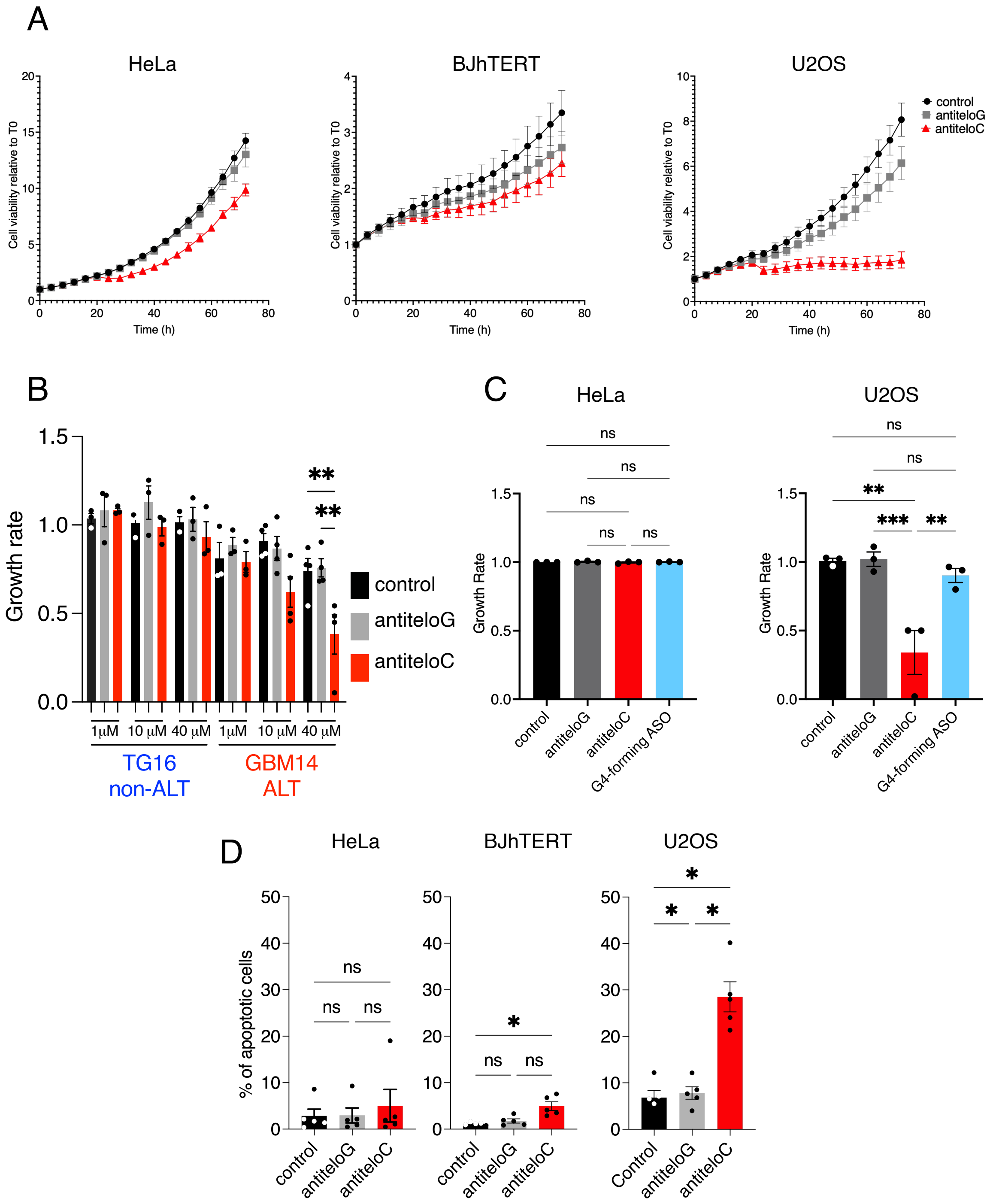

E

β-galactosidase

	control	antiteloG	antiteloC
Positive cells	0	0	0
Total cells	226	283	322

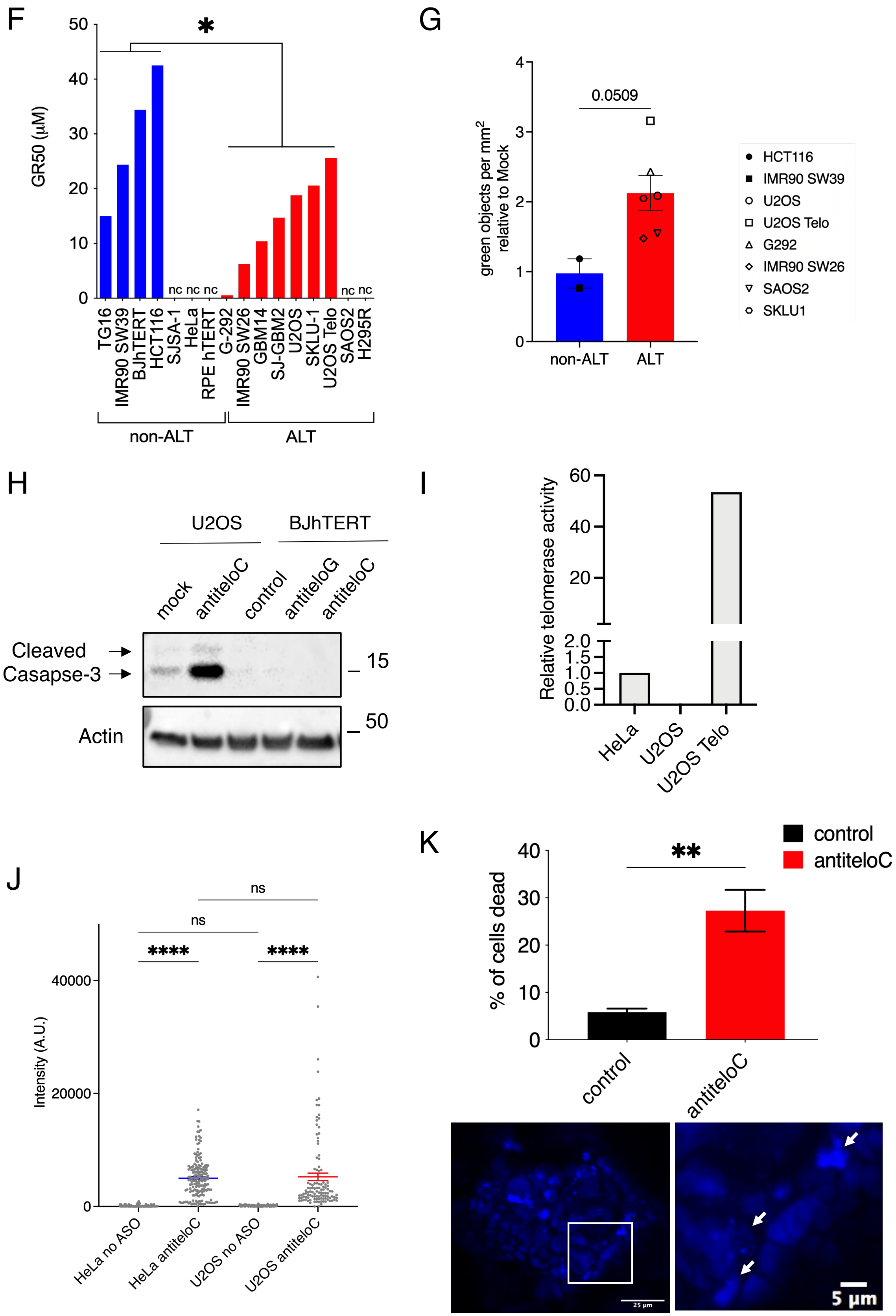
F
GR50 (µM)
50
40
30
20
10
0
*
nc nc nc
nc nc
TG16
IMR90 SW39
BJhTERT
HCT116
SJSA-1
HeLa
RPE hTERT
G-292
IMR90 SW26
GBM14
SJ-GBM2
U2OS
SKLU-1
U2OS Telo
SAOS2
H295R
non-ALT
ALT
G
green objects per mm2 relative to Mock
4
3
2
1
0
0.0509
non-ALT
ALT
HCT116
IMR90 SW39
U2OS
U2OS Telo
G292
IMR90 SW26
SAOS2
SKLU1
H
U2OS
BJhTERT
mock
antiteloC
control
antiteloG
antiteloC
Cleaved Casapse-3
15
Actin
50
I
Relative telomerase activity
60
40
20
2.0
1.5
1.0
0.5
0.0
HeLa
U2OS
U2OS Telo
J
Intensity (A.U.)
40000
20000
0
ns
ns
****
****
HeLa no ASO
HeLa antiteloC
U2OS no ASO
U2OS antiteloC
K
% of cells dead
40
30
20
10
0
**
control
antiteloC
control
antiteloC
25 µm
5 µm

L

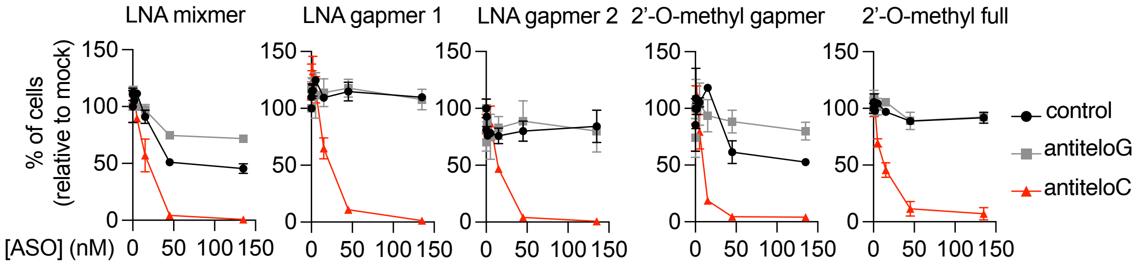

M

ASO	IC50 (nM)		
	control	antiteloG	antiteloC
LNA mixmer	nc	nc	8.56 (95%CI 6.35-11.52)
LNA gapmer 1	nc	nc	10.89 (95%CI 6.75-17.51)
LNA gapmer 2	nc	nc	10.33 (95%CI 6.97-15.25)
2'O-methyl gapmer	nc	nc	4.07 (95%CI 2.87-5.73)
2'O-methyl	nc	nc	7.46 (95%CI 5.19-10.69)

N

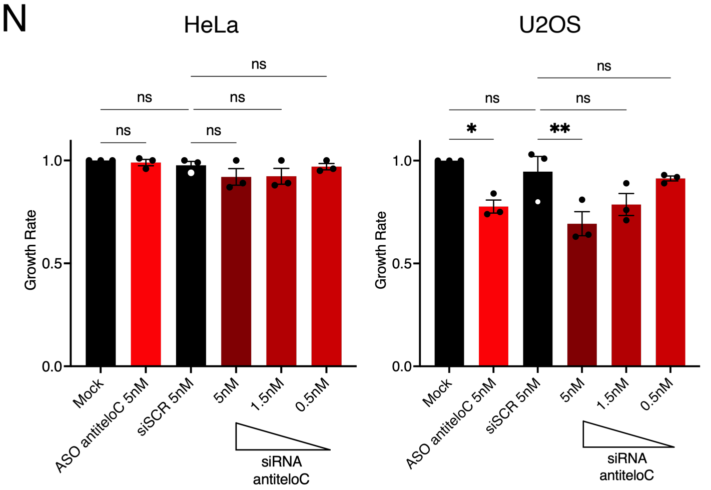

O

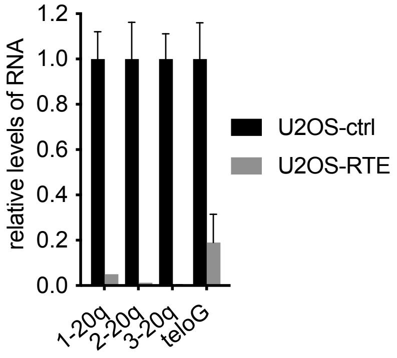

P

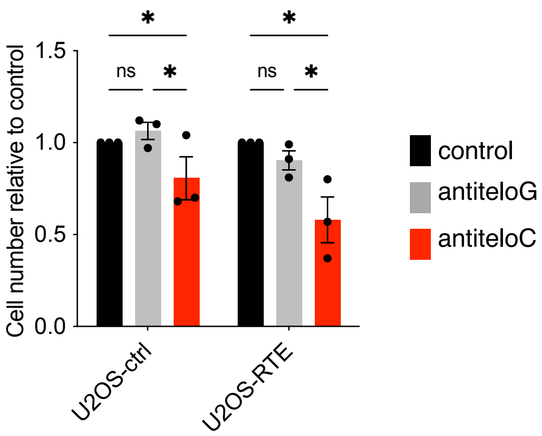

Supplementary figure 2: TeloC dilncRNAs are essential to maintain ALT cells viability. A Cells were treated with 10μM ASO without transfection reagent, and relative cells number was monitored in real-time by Incucyte for three days; data are presented as mean values +/- SD, n=2 technical replicates. **B** Cells were treated with ASO at the indicated concentrations without transfection reagent, and growth rate four days later was measured with resazurin; data are presented as mean values +/- SEM, n=3 biologically independent experiments for TG16 and 1μM ASO-GBM14, n=4 for 10μM ASO- and 40μM ASO-GBM14; one-way ANOVA, df=59, F=7.545, for control vs antiteloC **p= 0.0007; for antiteloG vs antiteloC **p= 0.0004. **C** Cells were transfected with 10nM ASO and growth rate three days later was measured with resazurin; data are presented as mean values +/- SEM, n=3 biologically independent experiments; one-way ANOVA, df=14, F(HeLa)=0.4813, F(U2OS)=13.51 ns=non-significant, for U2OS control vs antiteloC **p=0.0010, antiteloG vs antiteloC , ***p=0.0009, antiteloC vs G4-forming ASO **p=0.0037. **D** Cells were treated as in figure 2D where antiteloC ASO treatments shown here are directly compared; data are presented as mean values +/- SEM, n=5 biologically independent experiments; one-way ANOVA, df=24, F(U2OS)=21.77, F(BJhTERT)=17.67, F(HeLa)=1.809, for BJhTERt *p=0.0271; for U2OS control vs antiteloC *p=0.0205, control vs antiteloG *p=0.0436, antiteloG vs antiteloC *p=0.0209. **E** U2OS cells were transfected with 20nM ASO and fixed two days later for β-galactosidase analysis; n=2 biologically independent experiments. **F** Cells were treated with antiteloC ASO at a range of concentrations without transfection reagent and viability measured three days later by RealTime-Glo. GR_{50} values calculated accordingly, nc = not calculable due to lack of ASO response; significance calculated among n=4 non-ALT and n=7 ALT cell lines; two-tailed unpaired t-test, df=9, t=2.452, *p=0.0366. **G** Cells were treated with 10μM antiteloC ASO without transfection reagent and apoptotic cells (green objects) were counted by Incucyte three days after ASO delivery; data are presented as mean values +/- SEM, n=2 non-ALT and n=6 ALT cell lines; two-tailed paired t-test, df=6, t=2.433. **H** BJhTERT cells were treated with 10μM antiteloC ASO without transfection reagent and three days later analyzed by western blot; n=4 biologically independent experiments, one representative image shown. **I** Relative telomerase activity was calculated by qPCR with TRAPEZE Merck-Millipore; n=1. **J** Cells were treated with 10μM fluorescent ASO without transfection reagent and nuclear integrated fluorescent intensity was measured one day after ASO delivery; data are presented as mean values +/- SEM, n=3 biologically independent experiments; more than 100 cells were counted overall; one-way ANOVA, df=528, F=74.76, ns=non-significant, p<0.0001****. **K** Hoechst-stained cells in zebrafish larvae brain were analyzed for dead/dying-cell morphologies; data are presented as mean values +/- SEM, n=4 biologically independent experiments; more than 2300 cells were counted overall in 44 and 45 fish treated with control and antiteloC ASO, respectively; two-tailed unpaired t test, df=6, t=4.807, **p=0.003; bottom, example of staining and dead cell morphology; top, quantification. **L** U2OS cells were transfected with the indicated ASO at a range of concentrations and relative cells number was monitored in real-time by Incucyte for three days; data are presented as mean values +/- SD among n=2 technical replicates. **M** Table of calculated IC50 values of ASO tested in figure 2G, and figure S2J. **N** Cells were transfected with indicated amount of ASO or siRNA and growth rate was calculated based on cell viability values measured three days later with resazurin; data are presented as mean values +/- SEM, n=3 independent biological replicates; one-way ANOVA, df=29, F(HeLa)=2.309, F(U2OS) = 6.367, ns=non-significant,

*p=0.0277, **p=0.0093. **O** RNA was quantified by subtelomere-specific, or strand-specific RT-qPCR, and normalized on RPLP0; data are presented as mean values +/- SEM, n=3 technical triplicates. **P** Cells were transfected with 20nM ASO and relative cell number was measured three days later with resazurin; data are presented as mean values +/- SEM, n=3 independent biological replicates; two-way ANOVA, ns=non-significant, for U2OS-ctrl control vs antiteloC *p=0.0130, antiteloG vs antiteloC *p=0.0194; for U2OS-TRE control vs antiteloC *p=0.0130, antiteloG vs antiteloC *p=0.0194

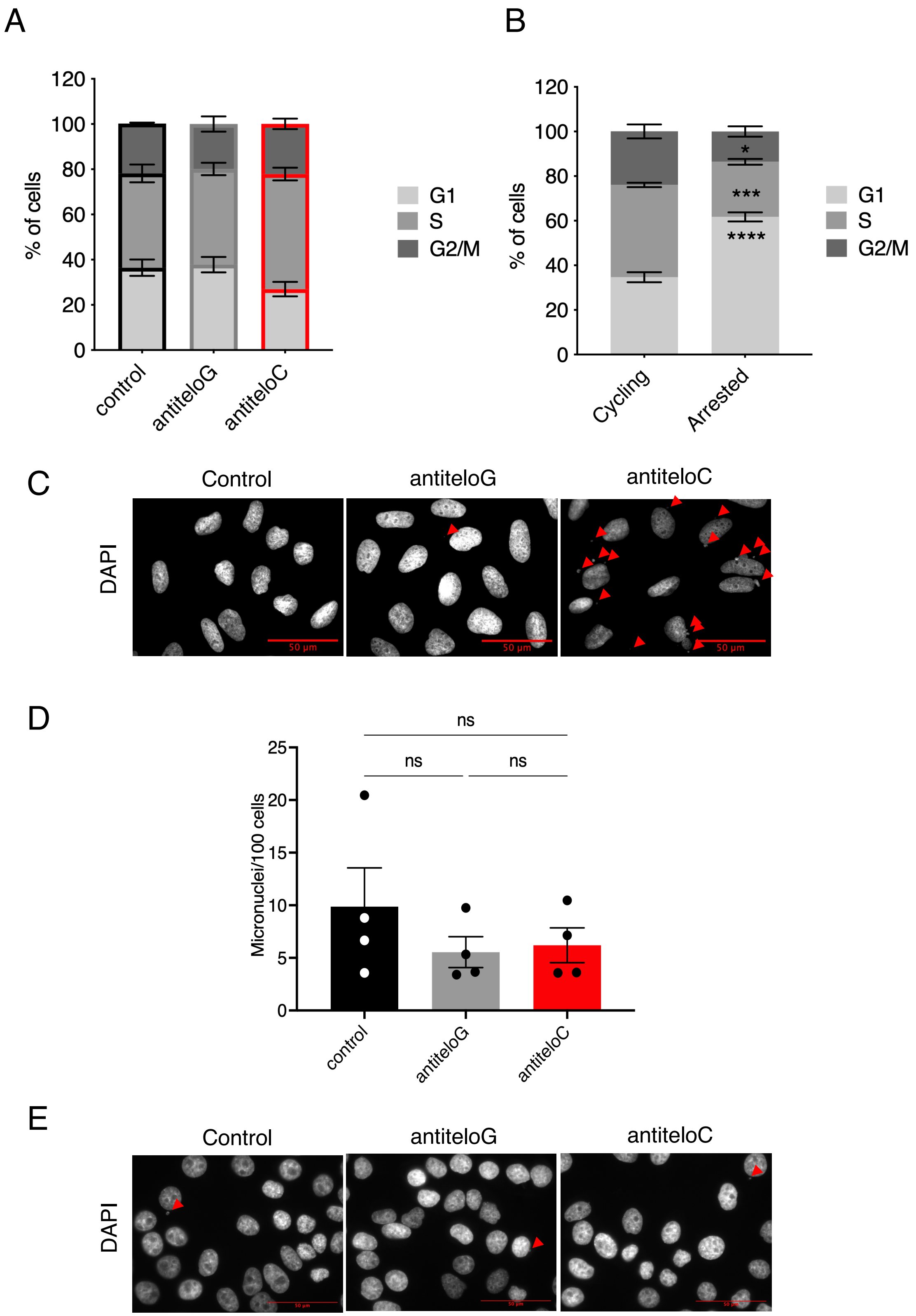
A
120
100
80
60
40
20
0
% of cells
control
antiteloG
antiteloC
G1
S
G2/M
B
120
100
80
60
40
20
0
% of cells
*
***
****
Cycling
Arrested
G1
S
G2/M
C
Control
antiteloG
antiteloC
DAPI
50 µm
50 µm
50 µm
D
ns
ns
ns
25
20
15
10
5
0
Micronuclei/100 cells
control
antiteloG
antiteloC
E
Control
antiteloG
antiteloC
DAPI

Supplementary figure 3: teloC dilncRNAs are required to cope with replication stress. A U2OS cells were transfected with 20nM ASO and cell cycle was analyzed two days later by flow cytometry; data are presented as mean values +/- SEM, n=4 biologically independent experiments. **B** SAOS2 cells were treated as in figure 3B and, one day after serum starvation, cell cycle was analyzed by flow cytometry; v data are presented as mean values +/- SEM, n=3 biologically independent experiments; two-way ANOVA, *p=0.0132, ***p=0.0004 <0.05*, ****p<0.0001. **C** Examples of staining from fig 3D. **D** HeLa cells were transfected with 20nM AS O and, two days later, fixed, stained with DAPI, and micronuclei manually counted; data are presented as mean values +/- SEM, n=4 biologically independent experiments; one-way ANOVA, df= 11, F=0.8840, ns=non-significant. **E** Examples of staining from D.

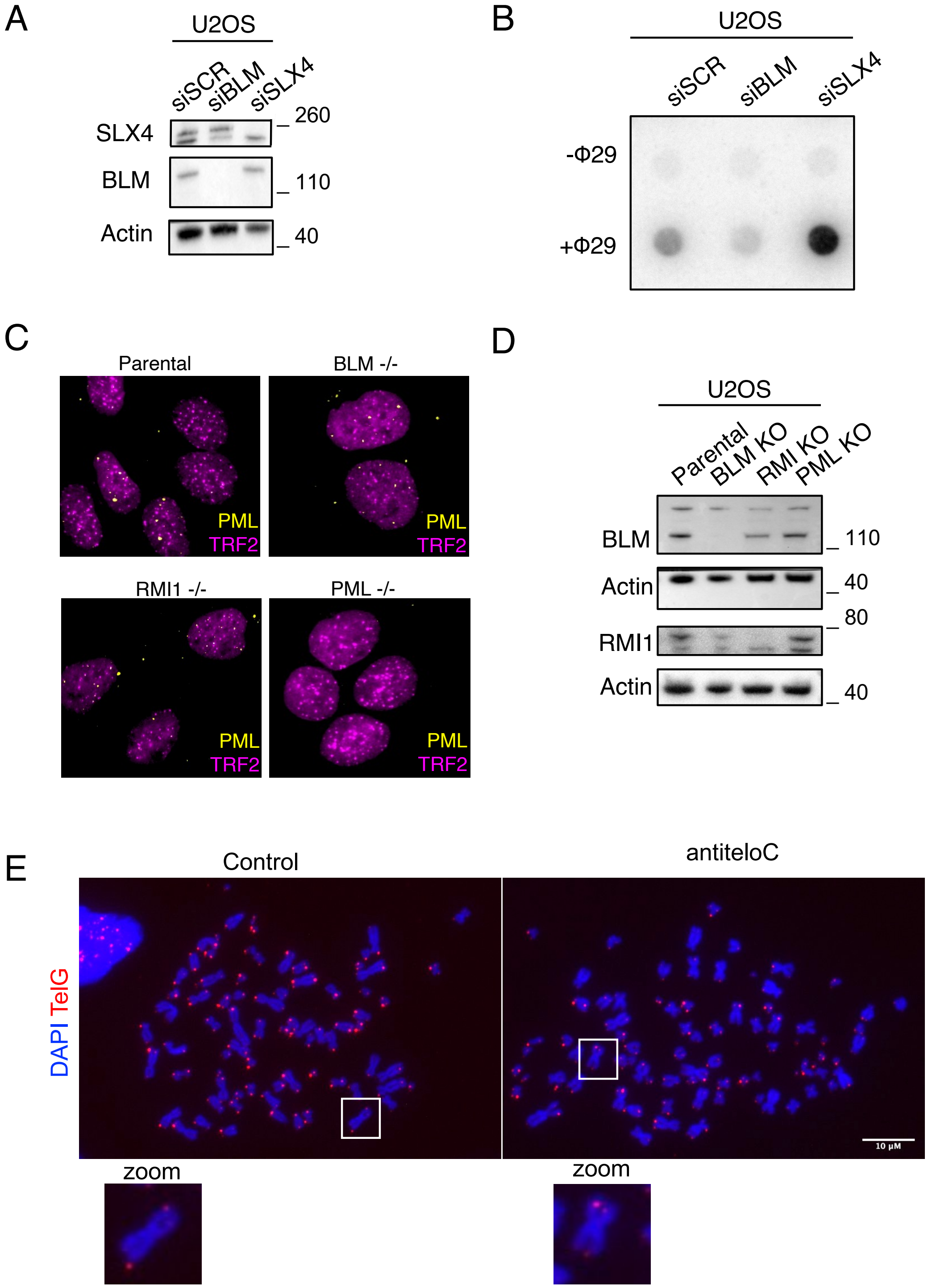
A
U2OS
siSCR
siBLM
siSLX4
SLX4
BLM
Actin
260
110
40
B
U2OS
siSCR
siBLM
siSLX4
-Φ29
+Φ29
C
Parental
BLM -/-
RMI1 -/-
PML -/-
PML
TRF2
D
U2OS
Parental
BLM KO
RMI KO
PML KO
BLM
Actin
RMI1
110
40
80
E
Control
antiteloC
DAPI TelG
zoom
10 μM

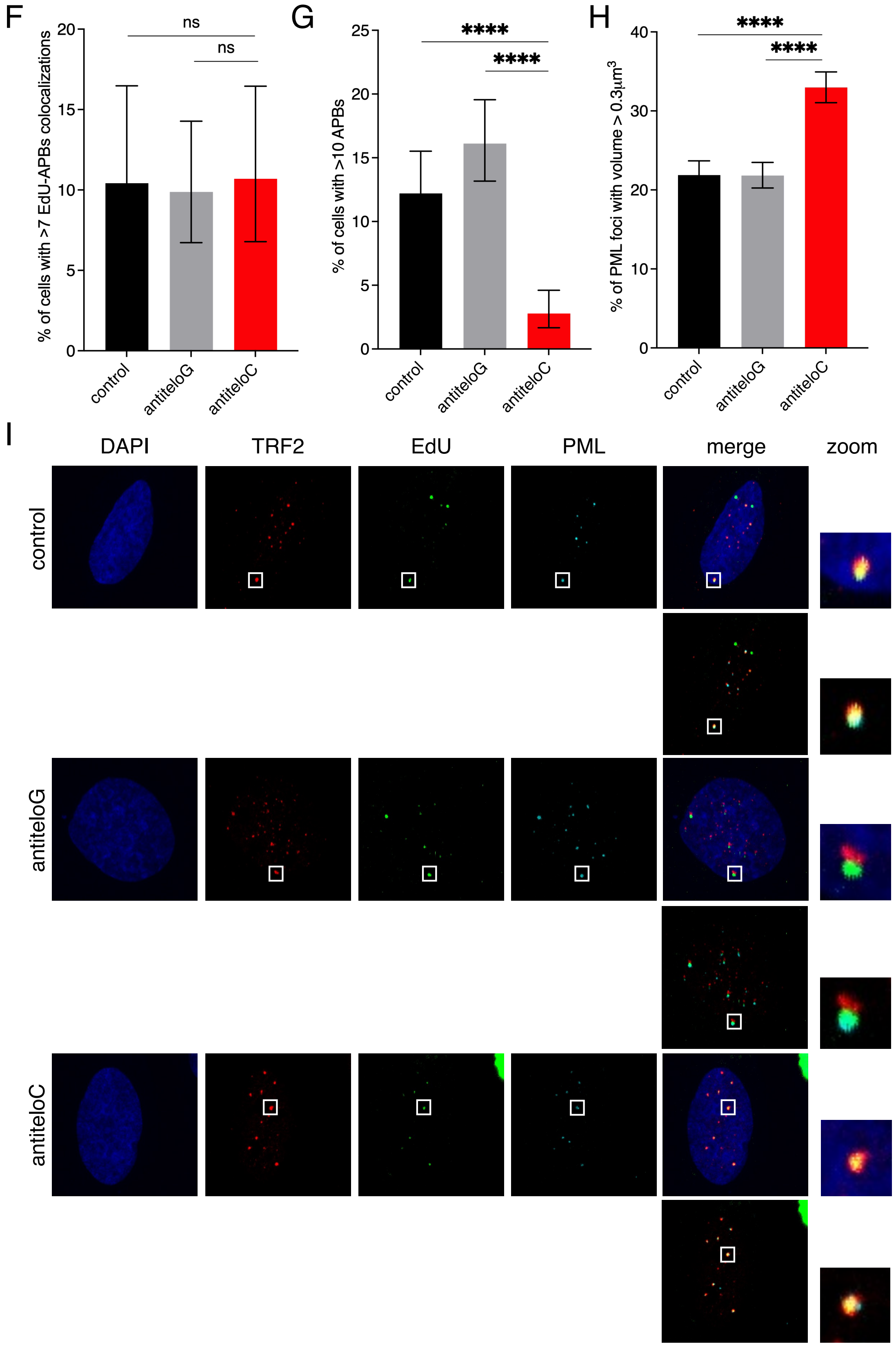
F
G
H
I
% of cells with >7 EdU-APBs colocalizations
% of cells with >10 APBs
% of PML foci with volume > 0.3µm3
ns
****
control
antiteloG
antiteloC
DAPI
TRF2
EdU
PML
merge
zoom

J

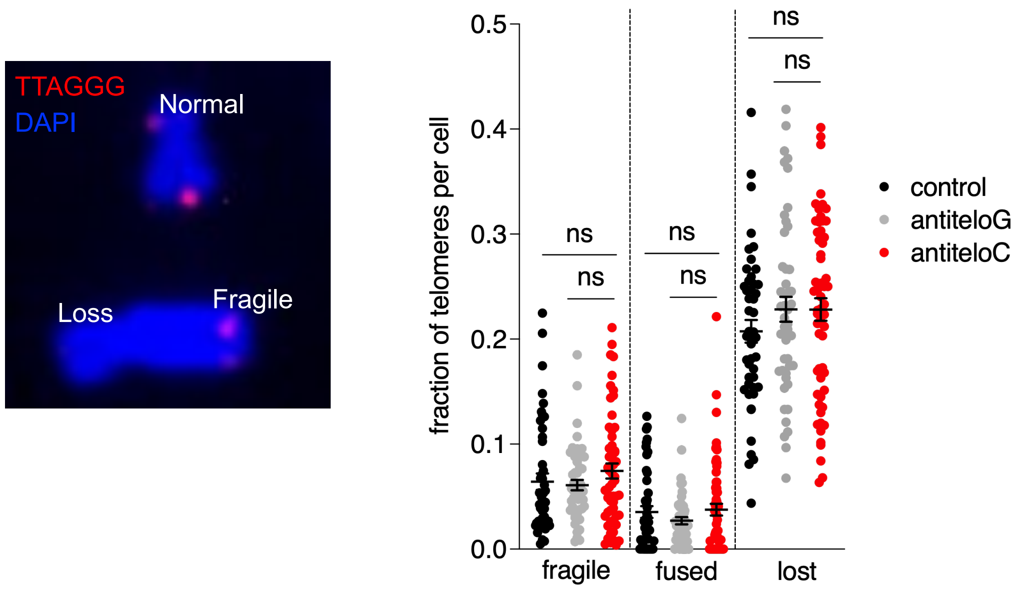

K

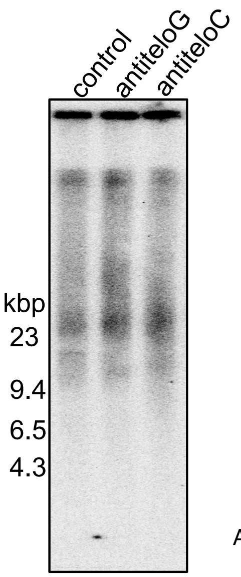

L

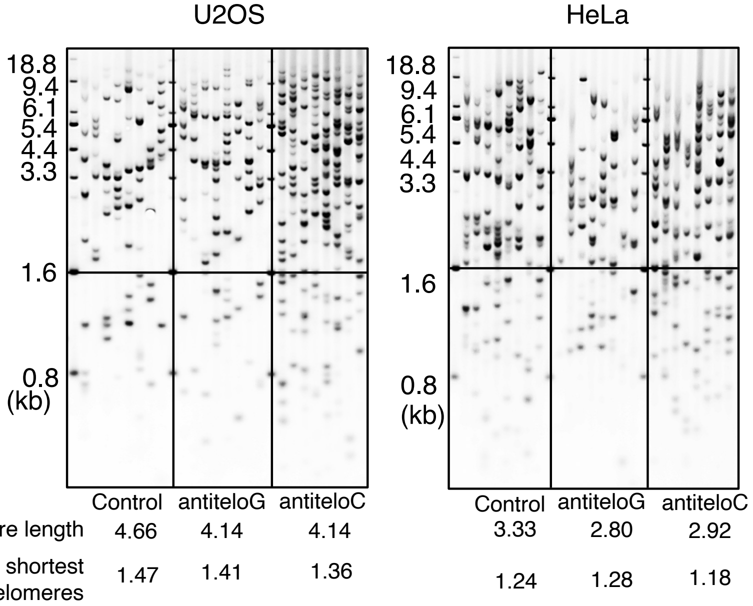

Supplementary figure 4: teloC dilncRNA inhibition upregulates unproductive break induced replication. A Western blot analysis of BLM and SLX4 from cells in figure 4A three days before viability measurements; n=3 biologically independent experiments, one representative image shown. **B** CCA from cells in figure 4A three days before viability measurements; n=3 biologically

independent experiments, one representative image shown. **C** Immunofluorescence staining for PML from cells in figure 4B, n=1 biologically independent experiment. **D** Western blot analysis of BLM and RMI1 from cells in figure 4B, n=1 biologically independent experiment. **E** Examples of staining from fig 4C. Zoom on the left shows a normal chromosome with two signals, zoom on the right shows a chromosome with three telomeric signals, indicating a T-SCE. **F-H** U2OS were transfected with 20nM ASO and pulsed for 2 h with 10μM EdU before fixation, two days after transfection. **F** Telomeric non-S DNA synthesis in APBs; data are presented as percentages ± 95% confidence interval, n=more than 140 cells over 3 biologically independent experiments; Fisher's exact test, ns=non-significant. Cells with less than 20 EdU foci were considered for quantification. **G** APBs quantification; data are presented as percentages ± 95% confidence interval, n=more than 450 cells over 3 biologically independent experiments; Fisher's exact test, p<0.0001****. **H** PML foci volumes quantifications; data are presented as percentages ± 95% confidence interval, n=more than 2000 PML foci over 3 biologically independent experiments; Fisher's exact test, p<0.0001****. **I** Examples of staining from fig 4E and S4F-H. Merges among DAPI, TRF2 and EdU on top; merges among TRF2, EdU and PML on bottom. **J** U2OS cells were transfected with 20nM ASO as indicated and, two days later, metaphases were stained with PNA probes; data are presented as mean values +/- SEM, n=more than 35 metaphases over 3 biologically independent experiments; one-way ANOVA, df= 147, F(fragile)=1.506, F(fused)=1.573, F(lost)=0.6757, ns=non-significant; left, example of staining and telomeric phenotypes analyzed, right, quantification. **K** U2OS cells were transfected with 20nM ASO and harvested two days later in agarose plugs. DNA was separated by pulsed-field gel electrophoresis, transferred to a membrane and hybridized with a radiolabeled telomeric probe. **L** U2OS and HeLa cells were transfected with the 20nM ASO as indicated, then genomic DNA was extracted and telomere length was analyzed by TeSLA.

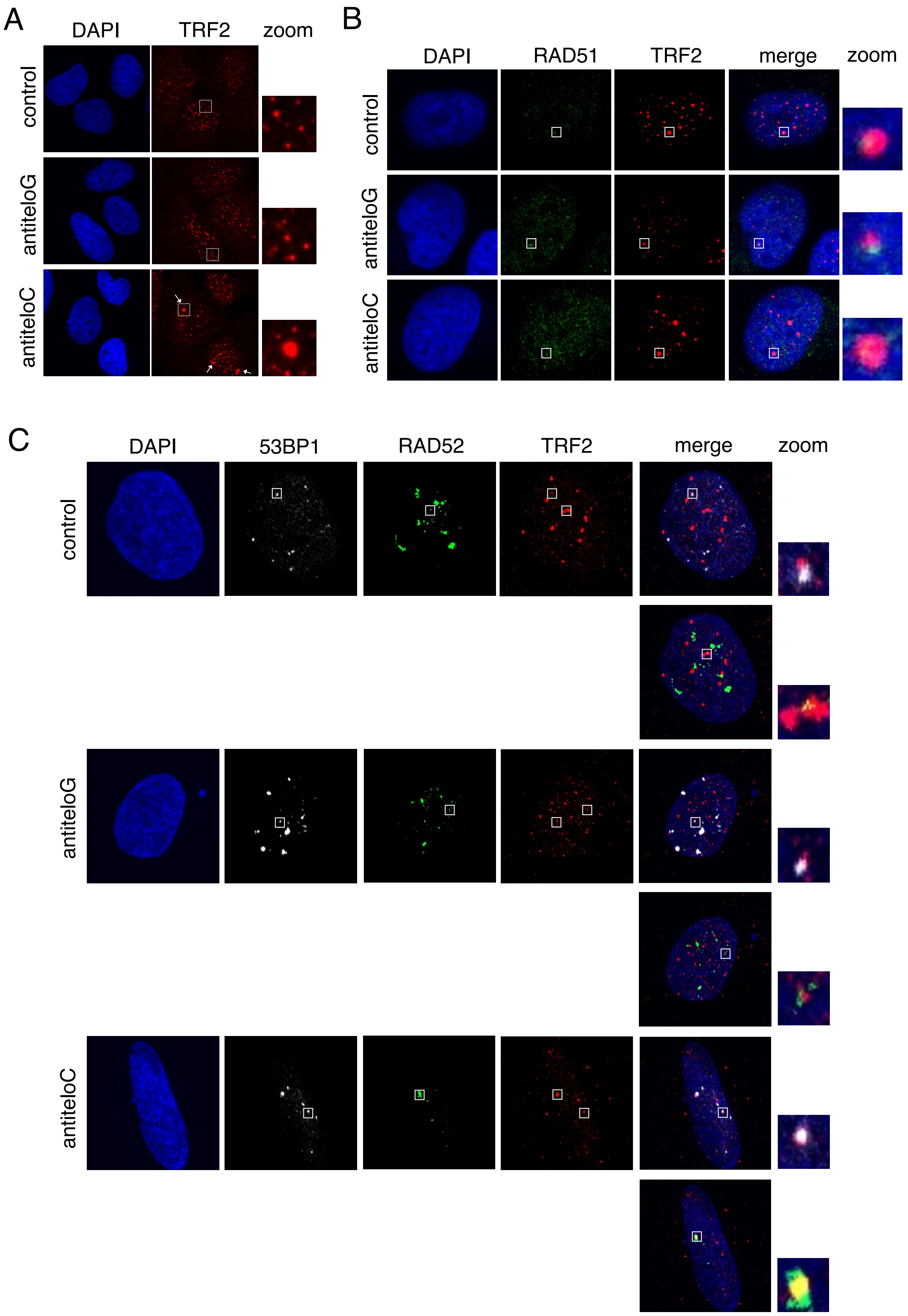
A
DAPI
TRF2
zoom
control
antiteloG
antiteloC
B
DAPI
RAD51
TRF2
merge
zoom
control
antiteloG
antiteloC
C
DAPI
53BP1
RAD52
TRF2
merge
zoom
control
antiteloG
antiteloC

D

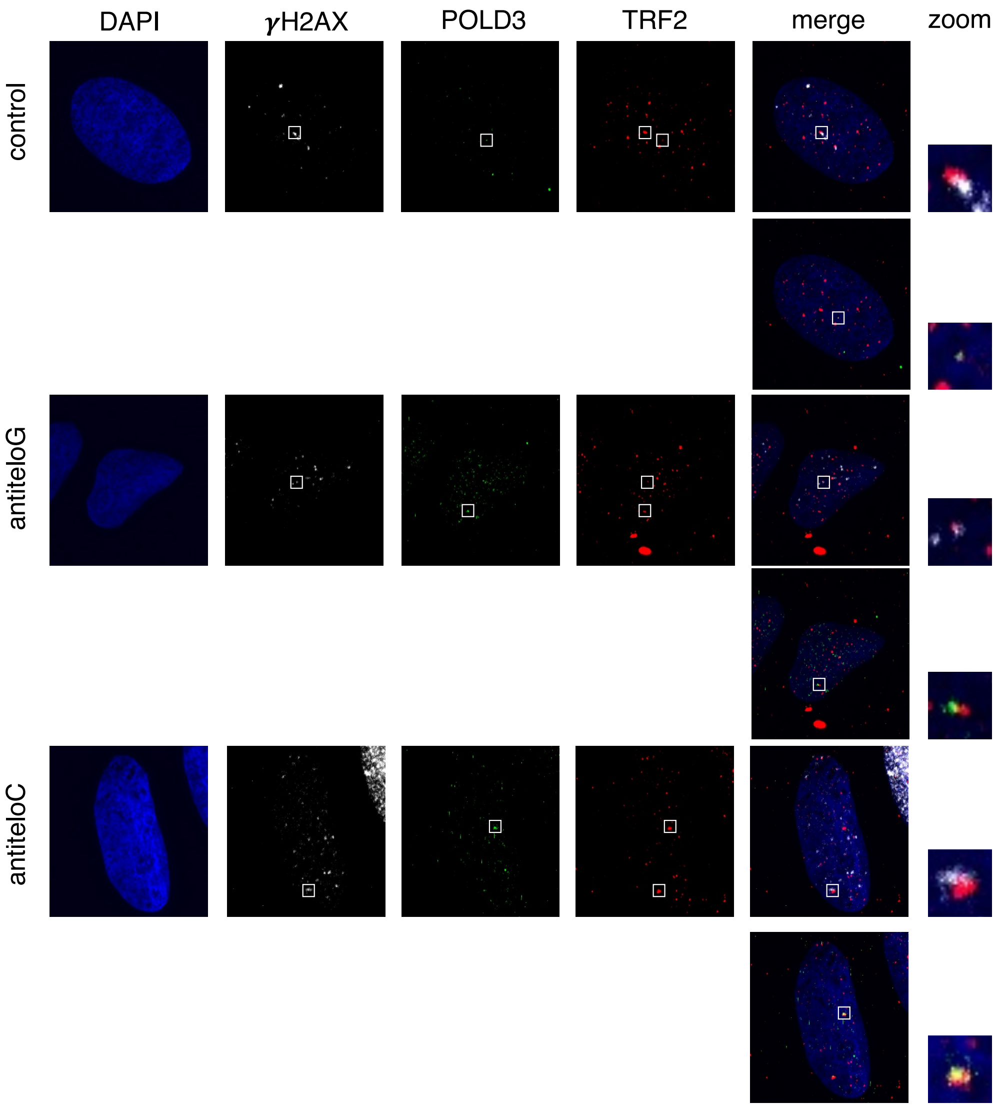
DAPI
γH2AX
POLD3
TRF2
merge
zoom
control
antiteloG
antiteloC

E

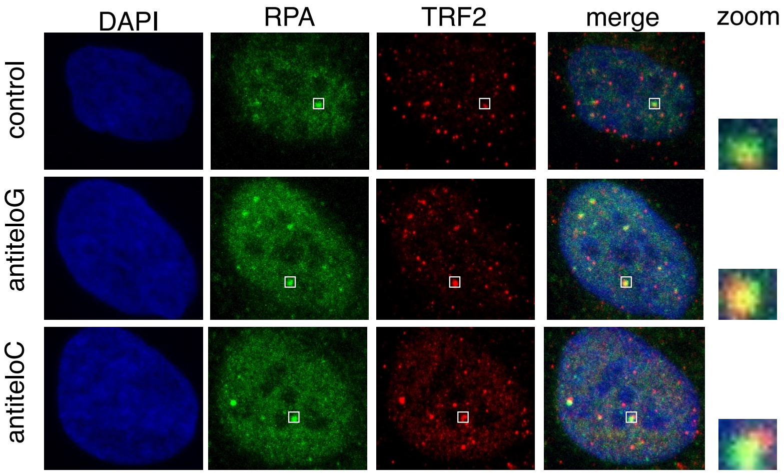
DAPI
RPA
TRF2
merge
zoom
control
antiteloG
antiteloC

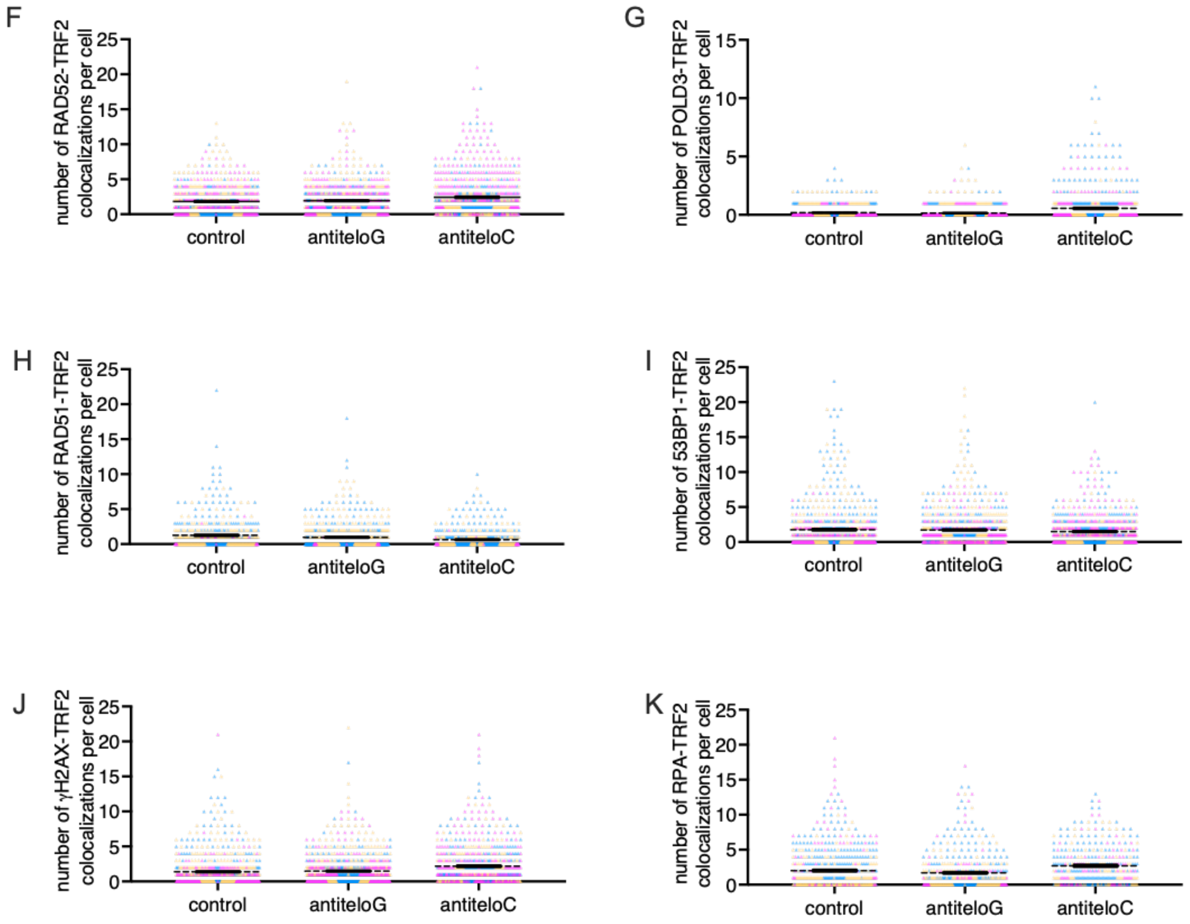

Supplementary figure 5: teloC dilncRNA inhibition alters the engagement of DDR factors at ALT telomeres. A Examples of staining from fig 5A. **B** Examples of staining from fig 5E. **C** Examples of staining from fig 5C and 5F. Merges among DAPI, TRF2 and 53BP1 are shown on top; merges among DAPI, TRF2 and RAD52 are shown on bottom. **D** Examples of staining from fig 5B and 5D. Merges among DAPI, TRF2 and γH2AX are shown on top; merges among DAPI, TRF2 and POLD3 are shown on bottom. **E** Examples of staining from fig 5G. **F-K** Different representation of data shown in figs. 5B-G, showing the number of colocalization events per cell between TRF2 and the indicated factors. Data are presented as mean values +/- SEM, dots in different colours are from the 3 biological independent experiments.
