# Supplementary material for: Alternative lengthening of telomeres (ALT) cells viability is dependent on C-rich telomeric RNAs
Source: Nat Commun. 2023 Nov 4;14:7086. doi: 10.1038/s41467-023-42831-0 (PMC10625592; doi:10.1038/s41467-023-42831-0)
Supplement: Supplementary file 2 — Reporting Summary [file 41467_2023_42831_MOESM2_ESM.pdf]

nature portfolio

Corresponding author(s): Fabrizio d'Adda di Fagagna

Last updated by author(s): Oct 6, 2023

Reporting Summary

Nature Portfolio wishes to improve the reproducibility of the work that we publish. This form provides structure for consistency and transparency in reporting. For further information on Nature Portfolio policies, see our Editorial Policies and the Editorial Policy Checklist.

Statistics

For all statistical analyses, confirm that the following items are present in the figure legend, table legend, main text, or Methods section.

n/a	Confirmed	
☐	☒	The exact sample size (*n*) for each experimental group/condition, given as a discrete number and unit of measurement
☐	☒	A statement on whether measurements were taken from distinct samples or whether the same sample was measured repeatedly
☐	☒	The statistical test(s) used AND whether they are one- or two-sided<br>*Only common tests should be described solely by name; describe more complex techniques in the Methods section.*
☒	☐	A description of all covariates tested
☒	☐	A description of any assumptions or corrections, such as tests of normality and adjustment for multiple comparisons
☐	☒	A full description of the statistical parameters including central tendency (e.g. means) or other basic estimates (e.g. regression coefficient) AND variation (e.g. standard deviation) or associated estimates of uncertainty (e.g. confidence intervals)
☐	☒	For null hypothesis testing, the test statistic (e.g. *F*, *t*, *r*) with confidence intervals, effect sizes, degrees of freedom and *P* value noted<br>*Give P values as exact values whenever suitable.*
☒	☐	For Bayesian analysis, information on the choice of priors and Markov chain Monte Carlo settings
☒	☐	For hierarchical and complex designs, identification of the appropriate level for tests and full reporting of outcomes
☒	☐	Estimates of effect sizes (e.g. Cohen's *d*, Pearson's *r*), indicating how they were calculated

Our web collection on statistics for biologists contains articles on many of the points above.

Software and code

Policy information about availability of computer code

Data collection	Images at widefield microscope were acquired with MetaMorph software; images at confocal microscope were collected with Leica Application Suite X;<br>Bio-Rad Image Lab 6.1 was used for immunoblot data collection;<br>Radioactive signal was acquired on a Typhoon (GE) scanner software version 2.0.0.6;<br>For flow cytometry studies see the related section.
Data analysis	For colocalization analysis, colocalizations events on the mosaic images were identified by a software-based analysis (Arivis Vision4D, v3.1.4). All the other digital images were analyzed in FIJI/ImageJ software (v1.53f51).<br><br>Bio-Rad Image Lab 6.1 was used for immunoblot data analysis.<br><br>Prism 9 software was used to generate graphs and to perform statistical analysis.

For manuscripts utilizing custom algorithms or software that are central to the research but not yet described in published literature, software must be made available to editors and reviewers. We strongly encourage code deposition in a community repository (e.g. GitHub). See the Nature Portfolio guidelines for submitting code & software for further information.

Data

Policy information about availability of data

All manuscripts must include a data availability statement. This statement should provide the following information, where applicable:

- Accession codes, unique identifiers, or web links for publicly available datasets
- A description of any restrictions on data availability
- For clinical datasets or third party data, please ensure that the statement adheres to our policy

Provide your data availability statement here.

Research involving human participants, their data, or biological material

Policy information about studies with human participants or human data. See also policy information about sex, gender (identity/presentation), and sexual orientation and race, ethnicity and racism.

Reporting on sex and gender	N.A.
Reporting on race, ethnicity, or other socially relevant groupings	N.A.
Population characteristics	N.A.
Recruitment	N.A.
Ethics oversight	N.A.

Note that full information on the approval of the study protocol must also be provided in the manuscript.

Field-specific reporting

Please select the one below that is the best fit for your research. If you are not sure, read the appropriate sections before making your selection.

☒ Life sciences ☐ Behavioural & social sciences ☐ Ecological, evolutionary & environmental sciences

For a reference copy of the document with all sections, see nature.com/documents/nr-reporting-summary-flat.pdf

Life sciences study design

All studies must disclose on these points even when the disclosure is negative.

Sample size	We did not use any criteria to determine the sample size. As much data as possible was collected depending on the nature of the experiments or in order to have statistical analysis
Data exclusions	In figures 4C and supplementary fig. 4J outliers were removed using the ROUT (Robust regression and Outlier removal) method with the Prism software. The exclusion criteria were pre-established and based in Prism software.
Replication	For all the experiments at least 3 independent replicates were performed unless differently stated in the figure legends.
Randomization	No randomization method was used. However, proper controls are present for each experiment.
Blinding	Investigators were not always blinded to group allocation during data collection and analysis because no subjective evaluations were required. However, quantifications in figure 4C and supplementary fig. 4J were performed blind. Moreover, quantification of colocalizations events in all panels of figure 5 and dupplementary figures 4F-H were identified by a software-based analysis (Arivis Vision4D, v3.1.4) using a custom pipeline. Foci size in figure 5A was measured with an automated pipeline in ImageJ.

Reporting for specific materials, systems and methods

We require information from authors about some types of materials, experimental systems and methods used in many studies. Here, indicate whether each material, system or method listed is relevant to your study. If you are not sure if a list item applies to your research, read the appropriate section before selecting a response.

Materials & experimental systems

n/a	Involved in the study	
☐	☒	Antibodies
☐	☒	Eukaryotic cell lines
☒	☐	Palaeontology and archaeology
☐	☒	Animals and other organisms
☒	☐	Clinical data
☒	☐	Dual use research of concern
☒	☐	Plants

Methods

n/a	Involved in the study	
☒	☐	ChIP-seq
☐	☒	Flow cytometry
☒	☐	MRI-based neuroimaging

Antibodies

Antibodies used	Actin (1:2000, A2228, Sigma-Aldrich), ASF1a (1:1000, 2990, Cell Signaling), ASF1b (1:1000, MA5-14836, Thermofisher), BLM (1:2000, ab476, Abcam), BrdU (1:5, 347580, BD Biosciences) cleaved Caspase-3 (9661, Cell Signaling, 1:50 for FACS, 1:1000 for immunoblot), H2AX (1:1000, 9718, Cell Signaling), PARP1 (1:1000, Serotec), PML (1:100, SC966, Santa Cruz), POLD3 (1:500, H00010714-M01, Abnova) RAD51 (1:400, Ab213-100, Abcam), RAD52 (1:200, SC365341, Santa Cruz), RMI1 (1:200, NB100-1720, Novus Biologicals), SLX4 (1:1000, A302-270A, Bethyl), TRF2 (1:500, N20, Santa Cruz or 1:500, NB-11057130, Novus Biologicals), Tubulin (1:2000, T5168, Sigma), 53BP1 (1:1000, A300-272A, Bethyl).
Validation	All antibodies were validated by the manufacturer and were previously used in peer reviewed works. Methods of validation and references to published application for all antibodies are all present into manufacturer dedicated website page of each indicated product.

Eukaryotic cell lines

Policy information about cell lines and Sex and Gender in Research

Cell line source(s)	HeLa, WI-38 and WI-38 VA13 (ATCC); BJ-hTERT (ATCC); U2OS (ATCC), HCT116 (DSMZ) and G-292 (ECACC); SAOS2 (DSMZ); IMR90 SW26 and SW39 (gift from J. Shay); RPE hTERT (ATCC); JFCF-6/T.1J/6B, JFCF-6/T.1J/1.3C, JFCF-6/T.1C, and JFCF-6/T.1D (gift from R. Reddel). SI14, SI24, 6C3, and 8G12 (gift from A. Decottignes); SJSA-1 (ATCC); SJ-GBM2 (COGcell); SKLU-1 (ICLC ECACC); GBM-14 (gift from C. G. Eberhart); NCI-H295R (ATCC); U2OS hTERT and control (gift from A. Decottignes); U2OS RTE and control (gift form M. blasco). U2OS parental and BLM, RMI1, and PML KO (gift from E. L. Denchi) TG16 (gift of F.Boussin).
Authentication	Cell lines were authenticated by STR profiling (GenePrint system, Promega).
Mycoplasma contamination	All cell lines are negative for mycoplasma.
Commonly misidentified lines (See ICLAC register)	No commonly misidentified cell lines were used.

Animals and other research organisms

Policy information about studies involving animals; ARRIVE guidelines recommended for reporting animal research, and Sex and Gender in Research

Laboratory animals	Zebrafish larvae (wild type strains AB and Tubingen), injected 2 days post fertilization, experiment concluded before 5 days post fertilization.
Wild animals	This study did not involve wild animals.
Reporting on sex	The sex was not considered.
Field-collected samples	This study did not involve samples collected from the field.
Ethics oversight	All animal experiments were performed in accordance with European guidelines and regulations, according to which no ethical approval is required for zebrafish larvae up to 5 days postfertilization.

Note that full information on the approval of the study protocol must also be provided in the manuscript.

Plants

Seed stocks: na

Novel plant genotypes: na

Authentication: na

Flow Cytometry

Plots

Confirm that:

- [ ] The axis labels state the marker and fluorochrome used (e.g. CD4-FITC).
- [x] The axis scales are clearly visible. Include numbers along axes only for bottom left plot of group (a 'group' is an analysis of identical markers).
- [x] All plots are contour plots with outliers or pseudocolor plots.
- [x] A numerical value for number of cells or percentage (with statistics) is provided.

Methodology

Sample preparation: Experiments were carried out either on cells fixed in 1% formaldehyde, washed, then fixed again in 75% ethanol or on cells fixed in ethanol 75%.

Instrument: Samples were acquired on a BD Facs Canto II.

Software: Analysis was performed using ModfitLT 3.0 software.

Cell population abundance: For caspase positive and cell cycle at least 8000 events were analyzed per sample. For BrdU positive cells, at least 500 events were analyzed per sample.

Gating strategy: Cell doublets were removed and living cells selection was based on forward and side scatter. Single cells were gated based on their SSC-A vs. FSC-A and SSC-A vs. SSC-H parameters. 488nm laser and 530/30 filter were used for FITC (caspase and BrdU); 670nm laser and 585/42 filter were used for PI.

- [ ] Tick this box to confirm that a figure exemplifying the gating strategy is provided in the Supplementary Information.
